# Assessing Amphiphilic ABAB Zn(II) Phthalocyanines with Enhanced Photosensitization Abilities in In Vitro Photodynamic Therapy Studies Against Cancer

**DOI:** 10.3390/molecules25010213

**Published:** 2020-01-04

**Authors:** Miguel Á. Revuelta-Maza, Marta Mascaraque, Patricia González-Jiménez, Arturo González-Camuñas, Santi Nonell, Ángeles Juarranz, Gema de la Torre, Tomás Torres

**Affiliations:** 1 Departments of Organic Chemistry and Biology, Universidad Autónoma de Madrid. C/Francisco Tomás y Valiente 7, 28049 Madrid, Spain; miguel.revuelta@uam.es (M.Á.R.-M.); marta.mascaraque@uam.es (M.M.); patri.gj.95@gmail.com (P.G.-J.); arturo.gonzalezc01@estudiante.uam.es (A.G.-C.); 2 Instituto Ramón y Cajal de Investigación Sanitaria, 28034 Madrid, Spain; 3 Institut Químic de Sarrià. Universitat Ramon Llull, 08017 Barcelona, Spain; santi.nonell@iqs.url.edu; 4 Instituto Madrileño de Estudios Avanzados (IMDEA)-Nanociencia. C/Faraday 9, Cantoblanco, 28049 Madrid, Spain

**Keywords:** Zn(II) phthalocyanines, ABAB, amphiphilic, photodynamic therapy, HeLa, SCC-13

## Abstract

We have previously demonstrated that singlet oxygen photosensitization abilities of Zn(II) phthalocyanines (Zn(II)Pcs) are enhanced through α-functionalization with bulky fluorinated substituents (i.e., bis(trifluoromethyl)phenyl units) at facing positions of ABAB Zn(II)Pcs, where A and B refer to differently functionalized isoindoles. In this work, we have prepared the Zn(II)Pc **ABAB 1** endowed with hydrophilic triethylene glycol monomethyl ether (i.e., at the A isoindoles) to provide solubility in aqueous media, together with its **A_3_B** and **A_4_** counterparts, and compared their ability to behave as photosensitizers for photodynamic therapy. All photophysical data, aggregation studies and preliminary in vitro biological assays in cell cultures of SCC-13 (squamous cell carcinoma) and HeLa (cervical cancer cells), have proved **ABAB 1** as the best photosensitizer of the series.

## 1. Introduction

Phthalocyanines (Pcs) stand out for their chemical stability, versatility, and propensity to generate reactive oxygen species (ROS) when photoexcited with light of the far red region of the UV–Vis spectrum [1]. These properties make Pcs interesting for their application in medicine as photosensitizers (PS) for photodynamic therapy (PDT) against cancer [2]. Owing to their extended π-conjugation, Pcs usually exhibit high aggregation tendency and solvophobic effects, forming oligomers in solution [3,4]. This high level of aggregation affects their photochemical and photophysical properties, which results in their photodynamic inactivation, avoiding ROS and singlet oxygen (^1^O_2_) generation and diffusion. Yet, aggregation tendency is not only their unique weakness, but also their inherent insolubility in water (which is crucial in order to reach the therapeutic target). These two facts together restrict drastically Pcs for in vivo application as PS. In this context, the choice of adequate substituents is decisive to achieve solubility in aqueous media and hinder their aggregation. Hydrophilic groups can be introduced to reach water solubility [5], whereas aggregation can be reduced or avoided by the introduction of bulky substituents in the Pc [6]. Among the different metallic Pcs used for PDT, Zn(II)Pcs stand out as one of the most promising, as they present high efficiencies in the generation of ^1^O_2_ [7,8,9,10]. Recently, our research group has described new paradigmatic ABAB Zn(II)Pcs (with A and B coding for differently functionalized isoindole constituents) that show both hindered aggregation and notably high ^1^O_2_ quantum yield (*Φ*_Δ_) values, featuring also chemical flexibility to incorporate additional functionalization that affords, for instance, water solubility [6]. For that purpose, a bulky phthalonitrile precursor endowed with bis(trifluoromethyl)phenyl units is used to avoid aggregation in the target ABAB Zn(II)Pc, but also to hamper its self-condensation, and thus force its cross-condensation with other phthalonitrile [11], which can be functionalized with hydrophilic groups.

The use of polyethylene glycol (PEG) chains is a popular approach to enhance the biocompatibility of hydrophobic drugs in general [12], and of PDT agents in particular. Among other advantages, PEG chains lead to a prolonged blood-circulating lifetime, allows for the minimization of nonspecific uptake and favors the enhanced permeability and retention (EPR) effect. All that features combined can result in an elevated concentration of the drug at the tumor site [13]. From a chemical point of view, PEG chains have proved to enhance the solubility in a variety of solvents, including nonpolar ones, aprotic polar solvents like DMSO, and protic polar solvents such as water [14,15]. Several Pcs functionalized with PEG chains of different length as peripheral [13,15,16,17] or axial groups [18,19] have shown solubility in biological media, and some of them have been studied as PS for PDT [20,21]. 

**Scheme 1:**
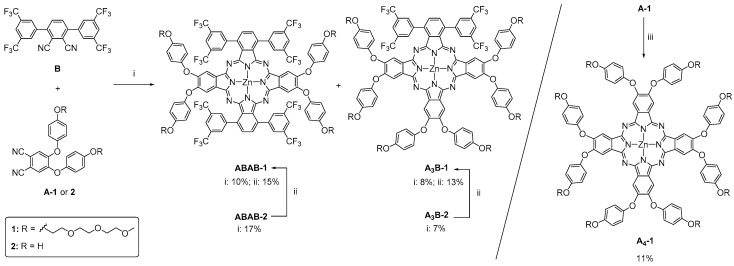
Synthesis of triethylene glycol monomethyl ether substituted **ABAB-1**, **A_3_B-1** and **A_4_-1** ZnPcs. (i) Zn(OAc)_2_, *o-*DCB/DMF (2:1), 150 °C, 15 h; (ii) TEG-Ts, K_2_CO_3_, DMF, 50 °C, overnight; (iii) Zn(OAc)_2_, pentanol, 1,8-diazabicyclo [5.4.0]undec-7-ene (DBU), 150 °C, 1h.

Herein, we report on the synthesis and characterization of a series of triethylene glycol (TEG)-containing Zn(II)Pcs, namely **ABAB-1, A_3_B-1** and **A_4_-1**, the study of their ^1^O_2_ generation capabilities, and preliminary biological assays to determine their potential as PS for PDT. The target Pcs have been synthesized following two different synthetic pathways: (i) a divergent route that allows to obtain generic ABAB and A_3_B Zn(II)Pcs functionalized with phenolic groups (**ABAB-2** and **A_3_B-2** Zn(II)Pcs in Scheme 1), which represent synthons, over which it is possible to add hydrophilic groups such as PEG chains; and (ii) the straightforward preparation of **ABAB-1** and **A_3_B-1** (and also **A_4_-1**) starting from adequately functionalized phthalonitriles. We have performed aggregation and photophysical studies in solution over this family of compounds in order to ratify our previous results regarding the enhanced ^1^O_2_ generation abilities of the nonaggregated ABAB-type Zn(II)Pc compounds with regard to their A_3_B and A_4_ counterparts [22]. Going one step further, we have performed in vitro assays to examine the toxicity of **ABAB-1**, **A_3_B-1** and **A_4_-1** in different tumor cells, i.e., SCC-13 and HeLa, as well as their cell localization. The final goal here is to demonstrate the benefits of bis(trifluoromethyl)phenyl-containing Zn(II)Pcs versus other substitution patterns.

## 2. Results and Discussion

### 2.1. Synthesis

The synthesis of Pcs containing different substituents is usually carried out by cyclotetramerization of appropriately substituted precursors through the condensation of two types of phthalonitriles in a statistical process. Starting from the same proportion of both precursors, a mixture of six products is expected, but by using bulky phthalonitrile **B [11]** with two bis(trifluoromethyl)phenyl units, only three Zn(II)Pcs (i.e., ABAB, A_3_B and A_4_) are usually obtained. For the preparation of our targeted Zn(II)Pcs, in particular **ABAB-1** and **A_3_B-1,** endowed with bulky groups over B isoindole rings and hydrophilic TEG substituents over the A positions, we have performed cross-condensation reactions with the bulky phthalonitrile **B**, and either **A-1**—already containing the TEG moieties—or 4,5-bis(4-hydroxyphenoxy)phthalonitrile **A-2 [23]** (see Scheme 1). The latter approach, yielding the intermediate Zn(II)Pcs **ABAB-2** and **A_3_B-2** [24] that should be further O-alkylated, was tackled in the basis of previous studies, which had shown that the crossed condensation between **B** and **A-2** is a highly efficient process (see below), and the Zn(II)Pcs formed are easily isolated from the reaction mixture [24].

Phthalonitrile **A-1** was thus prepared by *O-*alkylation of **A-2** [23] with the tosyl derivative of triethylene glycol monomethyl ether (TEG-Ts) [25] in 53% yield (see Scheme S1). Then, the reaction between phthalonitriles **A** and **B** was carried out by heating an equimolar mixture of them at 150 °C in *o-*dichlorobenzene (*o-*DCB)/DMF and in the presence of Zn(OAc)_2_. For the selected A:B ratio, the yield in A_3_B Zn(II)Pcs is lower (8% for **A_3_B-1** and 7% for **A_3_B-2**) than those obtained for their corresponding ABAB Zn(II)Pcs (10% for **ABAB-1** and 17%, for **ABAB-2**). The *O-*alkylation reaction of **ABAB-2** and **A_3_B-2** with TEG-Ts to give, respectively, **ABAB-1** and **A_3_B-1** took place in poor yields (15% and 13%, respectively), making this linear approach less efficient than the preparation of **A-1** and its condensation with **B**. It is worth mentioning that the corresponding symmetric **A_4_** Zn(II)Pc is not detected quantitatively in any reaction mixture. This symmetric octasubstituted, TEG-Zn(II)Pc **A_4_-1** is useful to perform a full comparative study of the influence of the bulky fluorinated substituents and the TEG moieties on the solubility, aggregation and ^1^O_2_ generation studies of this family of Zn(II)Pcs. In this context, this molecule was synthesized through a direct self-condensation of **A-1** phthalonitrile, using in this case the classical pentanol/DBU conditions, which gave rise to A_4_-1 in 11% yield.

**Figure 1:**
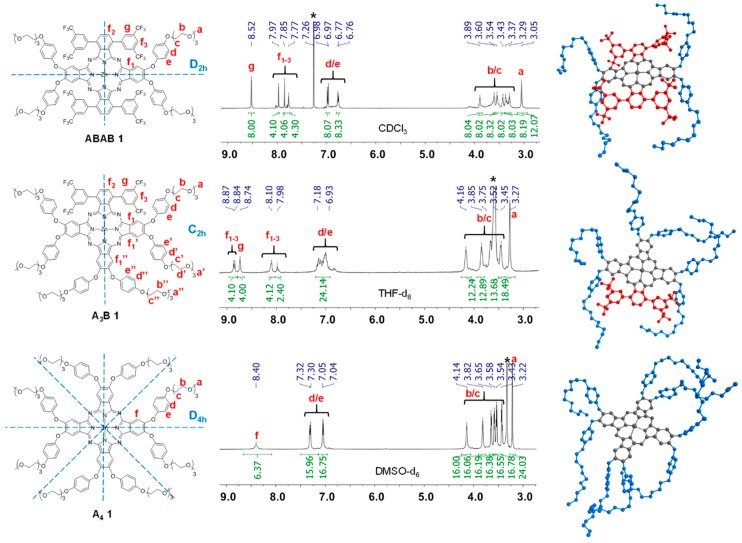
^1^H NMR spectra for **ABAB-1** (in CDCl_3_), **A_3_B-1** (in THF-d_8_) and **A_4_-1** (in DMSO-d_6_). Geometry optimization (MM2, SCIGRESS (FJ 2.8.1 EU 3.3.1)) for each molecule is shown.

Figure 1 shows the ^1^H NMR spectra of **ABAB-1**, **A_3_B-1**, and **A_4_-1**. The signal pattern for each compound arises from the molecular symmetry group these compounds belong to, namely, D_2h_ for **ABAB-1,** C_2h_ for **A_3_B-1** and D_4h_ for **A_4_-1**. Thus, highly symmetrical **A_4_-1** presents less signals than **ABAB-1**, which at the same time exhibits a simpler spectrum than **A_3_B-1**. The ^1^H NMR of **ABAB-1**, shows four singlets for the aromatic protons of the isoindole units and the trifluoromethylphenyl groups, two doublets for the phenoxy substituents, and a set of signals for the TEG chains with a chemical shift between 3.0 and 4.5 ppm. **A_3_B-1** produces six singlets for the isoindole units and the trifluoromethylphenyl substituents, and a multiplet that corresponds to the presence of three types of phenoxy groups. Figure 1 also shows structural models for the three compounds, where the molecular amphiphilic nature is represented in blue (hydrophilic zone) and yellow (hydrophobic zone). The products show good solubility in polar media, and, although they are no soluble directly in water due to the strong amphiphilic character, they are soluble in DMSO/water or MeOH/water (1:99) mixtures.

### 2.2. Photophysical Characterization

The UV–Vis spectra of **ABAB-1** and **A_3_B-1**, in both non-coordinating toluene and coordinating THF, show split *Q-*bands accompanied by the typical vibrational absorptions, although splitting is much more pronounced in **ABAB-1**, which is consistent with its D*_2h_* symmetry (Figures S1 and S2 and Table 1). Indeed, the absorption spectra of these compounds do not show any evidence of aggregation, as otherwise expected due to the presence of rather bulky substituents at the non-peripheral positions of the Pc core. Further confirmation of the lack of aggregation for these Zn(II)Pc results from absorption studies performed at a range of concentrations (between ~0.5·10^−6^ M – 5·10^−6^ M) (Figures S1 and S2), with an analysis of linear regression between the intensity of the *Q-*band and the concentration giving R^2^ values of ~0.999. **A_4_-1**, with a regular single *Q-*band in its UV–Vis spectrum, does not show either any evidence of aggregation in these solvents, probably because the phenyloxy substituents hamper π–π stacking of the Pc cores as well (Figures S1 and S2). Increasing the number of α-bis(trifluoromethyl)phenyl substituents produces a red-shift of the *Q-*band maximum, namely, from 682 nm for **A_4_-1** to 712 nm for **ABAB-1** in toluene solutions (Table 1). On the other hand, fluorescence studies have been also performed in toluene and THF solutions of **ABAB-1**, **A_3_B-1** and **A_4_-1,** showing an increase in the Stokes shift as the number of bis(trifluoromethyl)phenyl units attached to the Zn(II)Pc increases (Figure S3). Fluorescence quantum yield (*Φ*_F_) (Figure S4), and singlet (*τ_S_*) and triplet state (*τ_T_*) lifetimes (extracted from Time-Resolved Fluorescence -TRF- experiments in Figure S5) are included in Table 1. The results are consistent with those of non-aggregated Zn(II)Pcs, and show a modest solvent dependence. The mono-exponential fluorescence decay kinetics confirm that **ABAB-1**, **A_3_B-1**, and **A_4_-1** are in monomeric form in THF and toluene solutions, and the singlet excited-state lifetimes are similar for the two solvents.

The quantification of the ϕ_Δ_, for **ABAB-1**, **A_3_B-1**, and **A_4_-1** has been performed by two different methods. The first one entails the measurement of the photoinduced decomposition of 1,3-diphenylisobenzofuran (DPBF) in DMSO solutions, under the irradiation of the corresponding Zn(II)Pcs. The spectroscopic data obtained from these measurements are shown in Figure S6. For the three compounds, the decrease in the DPBF absorption was completely linear (R^2^ = 0.99), while no change was observed in the absorption of Zn(II)Pcs during the measurement, which evidences that photobleaching is not taking place under the irradiation conditions. **ABAB-1**, **A_3_B-1**, and **A_4_-1** showed *Φ*_Δ_ values of 93%, 85%, and 53%, respectively. These values should be considered with caution since indirect methods could be subjected to different variables, but are useful for comparative purposes. From those results we can infer that the larger the number of bulky fluorinated groups at α-positions, the better the ^1^O_2_ generation efficiency, as otherwise expected considering previous studies with related compounds [22], which show the important role of the bulky, electron-withdrawing bis(trifluoromethyl) groups in the ^1^O_2_ production_._ It is important to mention that aggregation is not considered to evaluate these results since the three compounds are non-aggregated in DMSO (see below). On the other hand, we have evaluated the *Φ*_Δ_ for **ABAB-1**, **A_3_B-1**, and **A_4_-1** via the direct detection of ^1^O_2_ phosphorescence at 1275 nm exciting at 355 nm, both in toluene and THF solutions (Figure S7 and Table 1). The *Φ*_Δ_ values show the same trend observed in the indirect measurements, namely, a parallel increase with the number of bulky fluorinated substituents and, indeed, *Φ*_F_ and *Φ*_Δ_ change concomitantly in the opposite direction. Overall, the measured *Φ*_Δ_ values are moderately higher than those reported for other amphiphilic Zn(II)Pcs, which confirms that the substitution pattern of this family of compounds improves the photophysical properties of the dye core, rendering them adequate for PDT applications. Importantly, we tried to perform measurements of ^1^O_2_ generation in water, but only residual signals were detected, which can be rationalized by the formation of aggregates (see below).

### 2.3. Aggregation Experiments

**Figure 2:**
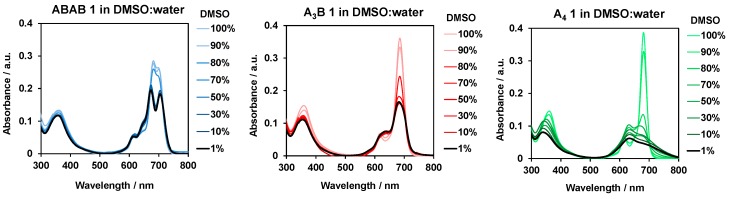
UV–Vis spectra of **ABAB-1**, **A_3_B-1**, and **A_4_-1** in different DMSO/water ratios.

Pcs have tendency to form aggregates from their aromatic systems for minimizing the solvation energy, particularly in the case of polar solvents as water. Although a certain organization of the dyes in vesicles or micelles can facilitate their transport through the blood system, it is important that once the PS is delivered to the target tissue, it remains as non-aggregated species, at least in some extent, that leading to more efficient ^1^O_2_ generation through their photoexcitation. For that reason, aggregation studies are important for the former evaluation of their potential application in vitro. Absorption and emission experiments were carried out over solutions of TEG-functionalized **ABAB-1**, **A_3_B-1**, and **A_4_-1**. Although they are not soluble in pure water, they appear to be soluble upon addition from stock DMSO solutions to Milli-*Q* water until a 1:99 DMSO/water ratio is reached, what is admissible for further in vitro assays. Therefore, we performed aggregation studies by registering UV–Vis and fluorescence spectra of the three Zn(II)Pcs in different mixtures of DMSO/water, in order to compare the specific behavior derived from the presence of two substituted isoindole units with bulky fluorinated groups (**ABAB-1**) versus one (**A_3_B-1**) or none of them (**A_4_-1**). Mixed solutions of DMSO/water ranging from 100:0 to 1:99 were measured, in both absorption (Figure 2) and emission (Figure S8) experiments. Importantly, initial measurements in pure DMSO correspond to a fully non-aggregated state for the three compounds. Looking at the absorbance spectra, we can determine that from 100% to 80% DMSO, **ABAB-1** presents an almost constant absorption for the *Q-*band, whereas **A_3_B-1** shows a gradual decrease of its intensity and broadening, as well as a rise of a band with shorter wavelength, compatible with the formation of co-facial *H-*type aggregated species. Furthermore, in the case of **ABAB-1**, when a 70:30 DMSO/water ratio is reached, the spectrum shows a sudden decrease of the *Q-*band intensity, and the concomitant emergence of a shoulder of at lower wavelength. Below 70% of DMSO contents, we can observe a constant behavior for both **ABAB-1** and **A_3_B-1** compounds, indicating that a stable situation is reached. In the case of **A_4_-1**, a sudden decrease of the *Q-*band, as well as a broadening and rising of a band at shorter wavelength compatible with the formation of aggregates, are observed starting at 80:20 DMSO/water ratios. In the case of emission studies, we can discern that the fluorescence of **A_3_B-1** and **A_4_-1** shows a sharp decrease in intensity when the water proportion increases, until a total cancellation of fluorescence at 70:30 DMSO/water ratio. On the other hand, **ABAB-1** shows a similar behavior than that observed in the absorption spectrum, that is, a huge decrease of the emission after the addition of 30% water. A magnification of this spectrum shows us that since this moment a residual fluorescence signal is kept with the same intensity, which means that we have reach a balanced situation with the presence of non-aggregated species in the medium.

### 2.4. Determination of the Log Po/w

**Figure 3:**
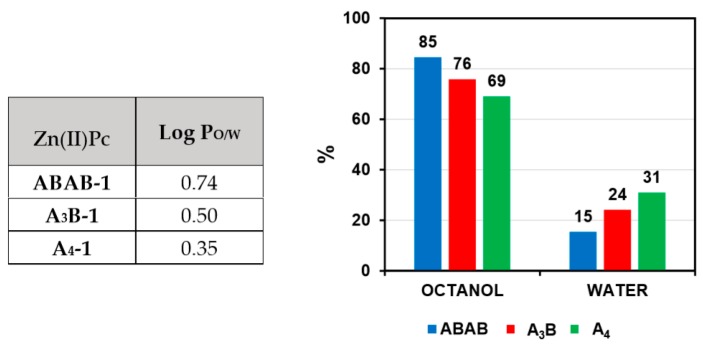
n-octanol/water partition experiments with **ABAB-1, A_3_B-1**, and **A_4_-1**.

For a preliminary evaluation of the relative lipophilicity of **ABAB-1**, **A_3_B-1**, and **A_4_-1**, and their affinity for cell membranes, *n*-octanol/PBS partition coefficients were determined using the shake-flask method (see Figure S9), which does not require standard compounds and is based on the direct determination of equilibrium partition concentrations of a compound in a biphasic system. Although the three compounds have a common structural Pc core, they exhibited different amphiphilic character. **ABAB-1** renders a higher octanol/water partition coefficient than **A_3_B-1** and **A_4_-1**, (see Figure 3), for [Zn(II)Pc] = 10^−5^ M in the octanol/water mixture (%DMSO < 1%), thus indicating the relative order of lipophilicity nature. In fact, the log P_O_/_W_ values suggest that **ABAB-1** and **A_3_B-1** are mostly hydrophobic and have very high affinity for membranes. On the other hand, **A_4_-1** is more amphiphilic, and its presence in the aqueous phase is more abundant, which is concordant which its higher solubility in this medium.

### 2.5. Biological Studies

Biological experiments with human HeLa (cervical adenocarcinoma cells) and SCC-13 (skin squamous carcinoma cells) strains have been performed using **ABAB-1**, **A_3_B-1**, and **A_4_-1** as PS, in order to evaluate the potential of our design.

#### 2.5.1. Cytotoxicity Studies

Phototoxicity of some Zn(II)Pcs has already been tested against cervical and oral skin squamous carcinoma cells in in vitro experiments. However, due to the aggregation problems they present, it is necessary to transport them through complex conjugates [26,27,28]. Herein, we have studied the activity of **ABAB-1**, **A_3_B-1** and **A_4_-1** Zn(II)Pcs as independent molecular entities upon red light irradiation by the 3-(4,5-dimethylthiazol-2-yl)-2,5-diphenyltetrazolium bromide (MTT) assay in SCC-13 and HeLa cell lines. For this purpose, we first evaluated the inherent toxicity of two different concentrations of the three Zn(II)Pcs (1·10^−6^ and 1·10^−7^ M) in cells, after 5 h of incubation in presence of PS in the dark. Separately, we have evaluated the possible damage caused by irradiation, treating the cells with the highest red light dose used in this work (9 J/cm^2^), in the absence of the PS. The results shown in Table 2 indicate that neither the presence of the Zn(II)Pc at these concentrations, nor the administration of red light, induced significant cytotoxic effects in the cell lines studied, and survival rates above 95% were obtained. In addition, we have analyzed whether the percentage of DMSO influences the single action of the PS or light, not observing changes in cell survival when combined with DMSO (Table 2).

Then, we evaluated the photodynamic activity of **ABAB-1**, **A_3_B-1**, and **A_4_-1** toward SCC-13 and HeLa cells upon irradiation. For the photodynamic treatments, we used two different concentrations (i.e., 1·10^−6^ and 1·10^−7^ M) and, after 5 h of incubation, cells were exposed to different red light doses (3, 6 and 9 J/cm^2^). It is important to mention here that, for a better comparison between the three compounds, one should calculate the total number of photons absorbed by each. However such calculation is very challenging, if not impossible, because they are likely to be aggregated to different extents, which leads to severe spectral changes (see Figure 2). Moreover, their distribution in the different cell compartments may be different, adding further uncertainty to the absorbance of the active species responsible for cell death. Thus, the only meaningful comparison is by concentration.

**Figure 4:**
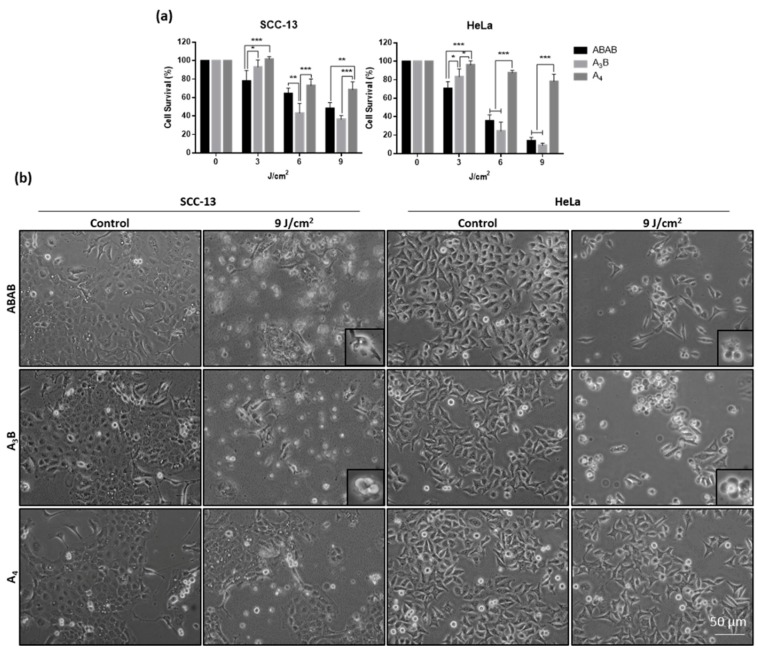
(**a**) Phototoxicity induced by **ABAB-1**, **A_3_B-1**, and **A_4_-1** in SCC-13 and HeLa cells. Cells were incubated with concentrations of 1·10^−6^ for 5 h and then irradiated with red light at variable doses. The survival was dependent of red light dose; **A_4_-1** was the less sensible to light irradiation. Cell survival was evaluated by the MTT test 24 h after treatments. Each value corresponds to the mean obtained from three independent experiments ± SD. (* *p* < 0.05; ** *p* < 0.01; *** *p* < 0.001). (**b**) Morphological changes observed in SCC-13 and HeLa cells 24 h after photodynamic treatment with the three Pcs (1·10^−6^ M, 5 h incubation followed by 9 J/cm^2^ of red light).

Figure S10 shows concentration-dependent response for the three compounds; cells were more sensitive to PDT when higher concentration (1·10^−6^ M) were applied. We selected the concentration of 1·10^−6^ M to perform the rest of experiments. Figure 4 shows the results for both cell lines treated with the Zn(II)Pcs; a drastic decrease in cell survival was revealed with the light dose in both cells lines. **ABAB-1** was the most sensitive at low red light dose, but the results for **ABAB-1** and **A_3_B-1** were similar when using 6 or 9 J/cm^2^ irradiations. Notably, the measured cell survival values are actually comparable to those of monomeric Si(IV)Pc-PEG species, which present IC_50_ values for HeLa cells of 0.28 μM [20], and also to other tetra-PEG functionalized Zn(II)Pcs [29]. On the other hand, **A_4_-1** was always less efficient in comparison with the other Zn(II)Pcs, leading to only 20% of cell death in both cell lines with the highest light dose (i.e., 9 J/cm^2^) (Figure 4a). Importantly, the trend found for cell survival values is parallel to the relative efficiency of the three Pc dyes as ^1^O_2_ generators (Table 1). Cell morphology of the treated cells was also analyzed 24 h after PDT using phase contrast microscopy (Figure 4b), revealing cytoplasmic retraction, with a rounded aspect similar to that of cells in apoptosis, in both cell lines treated with **ABAB-1** and **A_3_B-1** at higher light doses [30]. Interestingly, these images are well correlated with the results obtained from cell viability assays. We next analyzed by fluorescence microscopy the intracellular ROS formation in SCC-13 and HeLa cells when subjected to PDT with Zn(II)Pcs **ABAB-1**, **A_3_B-1** and **A_4_-1**, using for this purpose the DHF-DA fluorescent probe (Figure S11, Supporting Information). As shown in Figure S11, controls and cells incubated with Zn(II)Pcs in dark conditions did not exhibit green florescent due. In contrast, cells subjected to PDT showed an intense fluorescence in both cell types with the two of the compounds, **ABAB-1** and **A_3_B-1** (compared to baseline levels) revealing a prominent ROS production after PDT. However, cells treated with **A_4_-1** presented a very low fluorescence, indicating a low ROS production that is in agreement with the cell survival results.

#### 2.5.2. Localization Study

**Figure 5:**
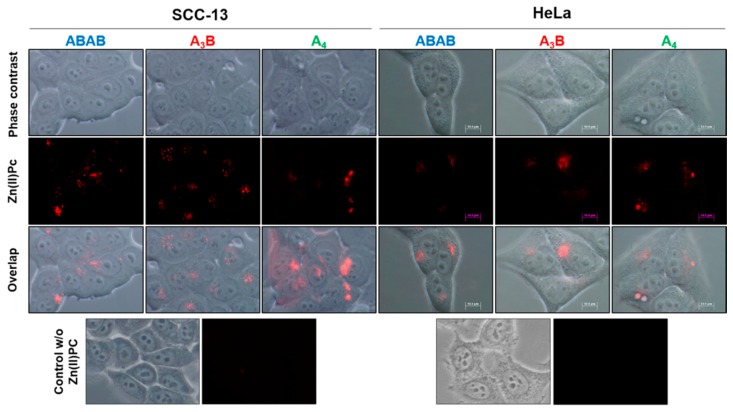
Cellular localization of **ABAB-1**, **A_3_B-1**, and **A_4_-1** (red) in SCC-13 and HeLa cells when they are observed with a fluorescence microscopy (irradiated with green light at 545 nm) and without other cell dye. They appear with a vesicular morphology inside cells.

**Figure A1:**
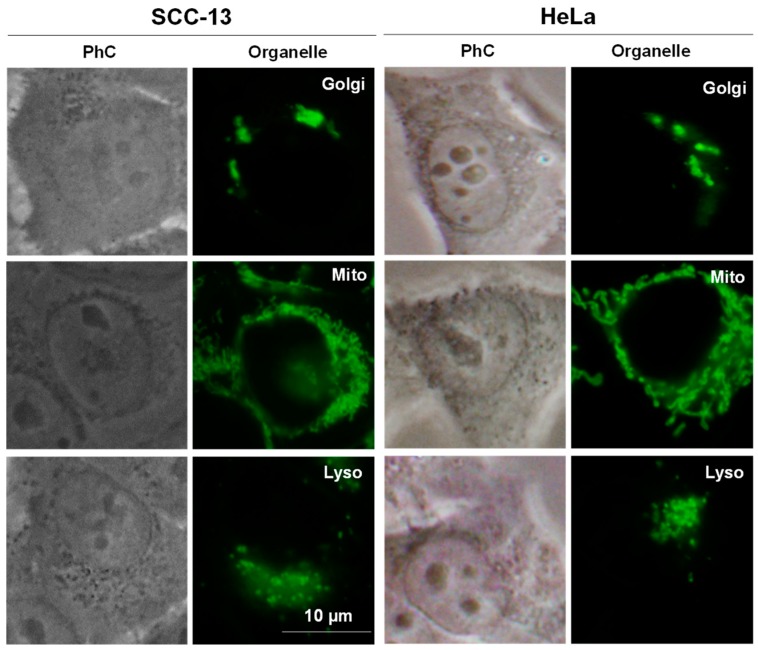
Subcellular localization of organelles of SCC-13 and HeLa cells after incubation with known fluorescent probes. Phase contrast (PhC). Green fluorescence is from Golgi apparatus (Golgi), mitochondrial (Mito) and lysosomes (Lyso). A blue (450–490 nm) exciting lamp was used for organelles probes. Scale bar 10 µm.

To localize the Zn(II)Pcs in the treated cells, they were irradiated with green light (545 nm) with a BP545 filter. In Figure 5 we can see that **ABAB-1**, **A_3_B-1**, and **A_4_-1** are located inside the cells. They appear with a vesicular morphology and they are probably localized in lysosomes or endosomes, which suggest that they are internalized through an endocytic pathway. Unfortunately, the fluorescence of these Zn(II)Pcs is too weak to be appreciated if specific probes for each organelle are used, which fluorescence mask the emission of the Zn(II)Pcs. For the sake of comparison, we are including in Appendix A (Figure A1) images of subcellular localization of organelles for SCC-13 and HeLa cells after incubation with known fluorescent probes. 

## 3. Materials and Methods

### 3.1. General Methods and Characterization Techniques

Chemical reagents were purchased from Merck-Sigma Aldrich, Alfa Aesar, Acros Organics, TCI or Fluorochem, as main commercial suppliers, and were used without further purification unless it is indicated. Solvents were purchased from Carlo Erba Reagents, and anhydrous solvents were dried with 4Å molecular sieves (Panreac). All reactions were performed in standard glasswares, except for the cyclotetramerization reactions that were carried out in high pressure sealed tubes. The monitoring of the reactions has been carried out by thin layer chromatography (TLC), employing aluminum sheets coated with silica gel type 60 F254 (0.2 mm thick, E. Merck). Purification and separation of the synthesized products was performed by column chromatography, using silica gel (230–400 mesh, 0.040–0.063 mm, Merck) and eluents are indicated for each particular case. Gel permeation chromatography (GPC) was performed using Bio-Beads S-X1 (200–400 mesh, Bio-Rad). Infrared (IR) spectra were recorded on a Cry 630 FTIR spectrophotometer from Agilent Technologies, using solid samples (diamond ATR). High-resolution mass spectrometry (HR-MS) were recorded using positive ESI Positive TOF_MS or matrix-assisted laser desorption/ionization (MALDI). Electrospray ionization (ESI) mass spectra were recorded with an API Q-Star Pulsar i from Sciex. Matrix used are indicated in each spectrum. MALDI-TOF HR-MS spectra were recorded with a Bruker Ultrareflex III spectrometer. MS data are expressed in *m/z* units. All MS experiments were carried out at the Servicio Interdepartmental de Investigación (SIdI) of the Universidad Autónoma de Madrid. Nuclear magnetic resonance spectra (^1^H NMR and ^13^C NMR) were recorded on Bruker AC-300 (300 MHz) or Bruker XRD-500 (500 MHz) instruments. Deuterated solvents employed are indicated in each spectrum. Aggregation studies were performed using aqueous miliQ-water, and organic spectroscopy grade solvents. UV–Vis spectra for aggregation experiments were recorded with a UV-vis JASCO-V660 spectrophotometer using 10 × 10 mm quartz cuvettes.

### 3.2. Synthesis

Compounds 3,3″,5,5″-tetrakis(trifluoromethyl)-[1,1′:4’,1″-terphenyl]-2′,3′-dicarbonitrile (**B**) [11], 4,5-bis(4-hidroxiphenoxy)phthalonitrile (**A-2**) [23], 2-(2-(2-methoxyethoxy)ethoxy)ethyl *p-*toluenesulfonate (**TEG-Ts**) [25], and **ABAB** Zn(II)Pc **2** [24], have been prepared according to published procedures.

*4,5-bis-(4-(2-(2-(2-methoxyethoxy)ethoxy)ethoxy)phenoxy)phthalonitrile* (**A-1**): In a two-neck round bottom flask, **A-2** (150 mg, 0.44 mmol) and **TEG-Ts** (291 mg, 0.91 mmol) were solved in dry DMF (6 mL) and heated to 50 °C. Then, anhydrous K_2_CO_3_ (199 mg, 1.44 mmol) was added in several portions. Reaction was kept at this temperature under argon atmosphere for three days. After cooling the reaction mixture was diluted with 100 mL of water and extracted with EtOAc (3 × 50 mL). The collected organic phase was washed with water (3 × 50 mL), brine (50 mL) and dried with MgSO_4_. The solvent was filtered and evaporated under reduced pressure. Then, product was purified by column chromatography on SiO_2_ with EtOAc as eluent, resulting in 275 mg (98% yield) of a brown oil. ^1^H NMR (300 MHz, CDCl_3_): *δ* 3.38 (s, 6H, TEG-CH_3_), 3.53–3.62 (m, 4H, TEG-CH_2_), 3.63–3.83 (m, 12H, TEG-CH_2_), 3.85–3.95 (m, 4H, TEG-CH_2_), 4.12–4.23 (m, 4H, TEG-CH_2_), 6.96–7.13 (m, 10H, CHAr). ^13^C NMR (75 MHz, CDCl_3_): *δ* 59.2, 68.2, 69.8, 70.7, 70.8, 71.0, 72.1, 109.9, 115.3, 116.5, 120.7, 121.6, 147.4, 152.6, 157.0. IR (ATR) ν^-1^ (cm^-1^): 2875, 2229, 1499, 1292, 1246, 1206, 1105. HR-MS (ESI+, +TOF, Ionizing phase: MeOH + NaI) for C_34_H_40_N_2_O_10_: *m/z* 659.2568 [M + Na]^+^, (calculated: 659.2575).

*Zn(II)Pc****A_3_B-2*:** Phthalonitrile **B** (150 mg, 0.27 mmol), phthalonitrile **A-2** (93 mg, 0.27 mmol) and anhydrous Zn(AcO)_2_ (50 mg, 0.27 mmol) were placed in a 5 mL high pressure resistant flask equipped with a magnetic stirrer, and then 2.7 mL ([**B**] = 0.1 M) of dry *o-*DCB/DMF (2:1) were added. The mixture was heated to 150–160 °C overnight under an argon atmosphere. After cooling the solvent was removed under vacuum. The product was purified by column chromatography on SiO_2_ (dioxane/heptane in gradient from 1:1 to 2:1) where the second fraction to elute containing the desired product **A_3_B-2**. The product was further purified by an additional column chromatography on Bio-Beads using CHCl_3_ as eluent. After evaporation of the solvent blue solid was obtained, which were recrystallized from dichloromethane (DCM)/heptane. Yield: 16 mg (7%). ^1^H NMR (300 MHz, DMSO-d_6_): *δ* 6.71–7.21 (m, 24H, O-Ph-O), 7.76 (s, 2H, CHAr), 8.04 (s, 2H, CHAr), 8.21 (s, 2H, CHAr), 8.63 (s, 4H, CHAr), 8.78 (s, 4H, CHAr), 9.28–9.56 (br s, 6H, OH). ^13^C NMR (75 MHz, THF-d_8_): *δ* 116.1, 116.4, 116.5, 118.8, 120.1, 120.9, 121.7, 125.3, 129.4, 129.8, 130.2, 131.1, 131.7, 133.4, 133.6, 134.4, 135.8, 142.5, 148.9, 149.0, 149.7, 150.1, 150.7, 150.8, 151.2, 151.2, 151.3, 153.1, 153.2, 153.5, 153.6, 153.9, 154.6, 154.9. IR (ATR) ν^-1^ (cm^-1^): 3416, 2954, 2883, 1723, 1507, 1449, 1362, 1279, 1196, 1182, 1133, 1034. MS (MALDI, matrix DCTB + PEGNa 1500) for C_84_H_44_F_12_N_8_O_12_Zn: *m/z* 1648.2177 [M]^+^, (calculated: 1648.2173).

*Zn(II)Pc **ABAB-1*****:** Method a**:** Phthalonitrile **B** (150 mg, 0.27 mmol), and **A-1** (172 mg, 0.27 mmol) and anhydrous Zn(AcO)_2_ (50 mg, 0.27 mmol) were placed in a 5 mL high pressure resistant flask equipped with a magnetic stirrer, and then 2.7 mL ([**B**] = 0.1 M) of dry *o-*DCB/DMF 2:1 were added. The mixture was heated to 150–160 °C overnight under an argon atmosphere. After cooling the solvent was removed under vacuum. Then, the product was purified by column chromatography on SiO_2_ (heptane/dioxane 1:1 as eluent) where the first green fraction to elute contained the desired product. It was further purified by an additional column chromatography on Bio-Beads using CHCl_3_ as eluent. After evaporation of the solvent a blue solid was obtained, which was recrystallized from DCM/heptane. Yield: 33 mg (10%). Method b**: ABAB**-**2** (10 mg, 0.0054 mmol) and **TEG-Ts** (34 mg, 0.11 mmol) were solved in dry DMF (7 mL) and heated to 50 °C. Then, anhydrous K_2_CO_3_ was added (7 mg, 0.048 mmol) and the reaction was kept under this temperature and argon atmosphere overnight. The reaction was monitored by TLC (heptane/dioxane) and extra anhydrous K_2_CO_3_ additions could be needed until complete the conversion. After cooling the solvent was evaporated under reduced pressure. Then, the product was purified by column chromatography on SiO_2_ (heptane/dioxane 1:1 as eluent). It was further purified by an additional column chromatography on Bio-Beads using CHCl_3_ as eluent. After evaporation of the solvent a blue solid was obtained, which was washed with heptane. Yield: 2 mg (15%). ^1^H NMR (500 MHz, CDCl_3_): *δ* 3.05 (s, 12H, TEG-CH_3_), 3.29 (m, 8H, TEG-CH_2_), 3.37 (m, 8H, TEG-CH_2_), 3.43 (m, 8H, TEG-CH_2_), 3.54 (m, 8H, TEG-CH_2_), 3.60 (m, 8H, TEG-CH_2_), 3.89 (m, 8H, TEG-CH_2_) 6.76 (d, 8H, *J =* 9.01 Hz, O-Ph-O), 6.98 (d, 8H, *J =* 9.01 Hz, O-Ph-O), 7.77 (s, 4H, CHAr), 7.85 (s, 4H, CHAr), 7.97 (s, 4H, CHAr), 8.52 (s, 8H, CHAr). ^13^C NMR (125 MHz, CDCl_3_): *δ* 58.8, 67.6, 69.7, 70.2, 70.4, 70.7, 71.8, 114.5, 115.5, 118.7, 121.5, 123.6 (q, *J =* 271.6 Hz, CF_3_); 131.2, 131.5, 134.8, 135.3, 136.8, 142.6, 151.1, 151.9, 152.4, 153.4, 154.6. IR (ATR) ν^-1^ (cm^-1^): 2916, 2874, 1501, 1410, 1377, 1275, 1198, 1178, 1127, 1104, 896. HR-MS (MALDI, matrix: DCTB + PPGNa 2000) for C_116_H_96_F_24_N_8_O_20_Zn: *m/z* 2440.5741 [M]^+^, (calculated: 2440.5644).

*Zn(II)Pc **A_3_B-1*****:** Method a**:** Phthalonitrile **B** (150 mg, 0.27 mmol), and **A-1** (172 mg, 0.27 mmol) and anhydrous Zn(AcO)_2_ (50 mg, 0.27 mmol) were placed in a 5 mL high pressure resistant flask equipped with a magnetic stirrer, and then 2.7 mL ([**B**] = 0.1 M) of dry *o-*DCB/DMF 2:1 were added. The mixture was heated to 150–160 °C overnight under an argon atmosphere. After cooling the solvent was removed under vacuum. Then, the product was purified by column chromatography on SiO_2_ (heptane/dioxane in gradient from 100:0 to 0:100) where the second green fraction to elute contained the desired product. It was further purified by an additional column chromatography on Bio-Beads using CHCl_3_ as eluent. After evaporation of the solvent a blue solid was obtained, which was recrystallized from DCM/heptane. Yield: 27 mg (8%). Method b**: A_3_B-2** (10 mg, 0.0061 mmol) and excess of **TEG-Ts** (39 mg, 0.12 mmol) were solved in dry DMF (7 mL) and heated to 50 °C. Then, K_2_CO_3_ (7.5 mg, 0.054 mmol) was added, and the reaction was kept under this temperature and argon atmosphere overnight. The reaction was monitored by TLC (heptane/dioxane) and extra anhydrous K_2_CO_3_ additions could be needed until complete the conversion. After cooling the solvent was evaporated under reduced pressure. Then, the product was purified by column chromatography on SiO_2_ (heptane/dioxane in gradient from 100:0 to 0:100). It was further purified by an additional column chromatography on Bio-Beads using CHCl_3_ as eluent. After evaporation of the solvent a blue solid was obtained, which was recrystallized from EtOAc/heptane. Yield: 2 mg (13%). ^1^H NMR (300 MHz, THF-d_8_): *δ* 3.27 (br s, 18H, TEG-CH_3_); 3.45 (br s, 12H, TEG-CH_2_), 3.52–3.75 (m, 36H, TEG-CH_2_); 3.85 (br s, 12H, TEG-CH_2_); 4.16 (br s, 12H, TEG-CH_2_); 6.93–7.18 (m, 24H, O-Ph-O); 7.98 (br s, 2H, CHAr); 8.10 (br s, 4H, CHAr); 8.74 (s, 4H, CHAr); 8.84 (s, 2H, CHAr); 8.87 (s, 2H, CHAr). ^13^C NMR (75 MHz, THF-d_8_): *δ* 59.0, 69.0, 70.8, 71.5, 71.7, 71.8, 74.6, 116.0, 116.2, 116.6, 116.8, 119.1, 119.6, 120.3, 120.4, 122.2, 123.8, 131.7, 132.0, 132.2, 133.2, 135.6, 135.8, 135.9, 136.0, 136.1, 136.6, 137.5, 138.3, 152.3, 153.4, 156.0, 156.5. IR (ATR) ν^−1^ (cm^−1^): 2870, 1501, 1450, 1404, 1276, 1198, 1127, 1093, 1029, 890, 800. HR-MS (MALDI, matrix: DCTB + PPGNa 2000) for C_126_H_128_F_12_N_8_O_30_Zn: *m/z* 2524.7894 [M]^+^, (calculated: 2524.7831).

*Zn(II)Pc **A_4_-1*****:** A mixture of **A-1** (90 mg, 0.14 mmol) and Zn(OAc)_2_ (9.7 mg, 0.053 mmol) was placed in a 5 mL high pressure resistant flask equipped with a magnetic stirrer, and then 1.4 mL of pentanol and 0.1 mL of DBU were added. Then mixture was heated to 150 °C under argon atmosphere for 1 h. After cooling the solvent was removed under vacuum. The product was purified by column chromatography on SiO_2_ (EtOAc/MeOH in gradient from 100:0 to 0:100). After evaporate the solvent under reduced pressure, the product was washed with DCM/heptane and a green solid was obtained. Yield: 9.7 mg (11%). ^1^H NMR (500 MHz, DMSO-d_6_): *δ* 3.22 (s, 24H, TEG-CH_3_); 3.43 (t, 16H, *J =* 5.05 Hz, TEG-CH_2_); 3.54 (t,16H, *J =* 5.05 Hz, TEG-CH_2_); 3.58 (t, 16H, *J =* 4.94 Hz, TEG-CH_2_); 3.65 (t, 16H, *J =* 4.94 Hz, TEG-CH_2_); 3.82 (br t, 16H, TEG-CH_2_); 4.14 (br t, 16H, TEG-CH_2_); 7.04 (d, 16H, *J =* 8.70 Hz, O-Ph-O); 7.31 (d, 16H, *J =* 8.70 Hz, O-Ph-O); 8.40 (br s, 3H, CHAr-Pc(core)). ^13^C NMR (125 MHz, DMSO-d_6_): *δ* 58.0, 67.6, 69.1, 69.6, 69.9, 70.0, 71.3, 112.3, 115.7, 119.8, 133.2, 149.8, 150.4, 154.9. IR (ATR) ν^−1^ (cm^−1^): 3016, 2933, 1502, 1450, 1425, 1400, 1284, 1200, 1130, 936. HR-MS (MALDI, matrix DCTB + PPGNa 2000 + PPGNa 2700) for C_136_H_160_N_8_O_40_Zn: *m/z* 2608.9966 [M]^+^, (calculated: 2609.0018).

### 3.3. Spectroscopic Techniques

*Φ*_Δ_ in DMSO was measured following an indirect method based on the photoinduced decomposition of a scavenger, in this case DPBF which reacts with ^1^O_2_ after the excitation with visible light filtered below 530 nm (hν > 530 nm) [31]. Non-substituted Zn(II)Pc was used as reference compound which present a *Φ*_Δ_ = 0.67 in DMSO [32]. For these studies, a stock solution in DMSO of DPBF was prepared (with an absorbance of ~1 a.u.), and 2 mL were transferred into a 10 × 10 mm quartz optical cell and bubbled with ^3^O_2_ for 1 min. Then, concentrated stock solutions of the corresponding Zn(II)Pcs in DMSO were added, in a defined amount to reach an absorbance of the final solution in the *Q-*band maximum of 0.1 ± 0.05 a.u. The solutions were stirred and irradiated for defined time intervals using a halogen lamp of 300 W. The length of time intervals was modified for each experiment, obtaining an absorbance diminution of DPBF ~3–4%. Incident light was filtered through a water filter (6 cm) and an additional filter to remove light under 530 nm (Newport FSQ-OG530 filter). Moreover, neutral density filters (FBS-ND03 and FB-ND10, were used). Decrease of DPBF absorbance with irradiation time was monitored at 414 nm and all experiments were performed three times. Average values are represented, and *Φ*_Δ_ is calculated through Equation (1); *R* and *S* indicate reference and sample, respectively. *k* is the slope of a plot of ln (*A_0_/A_t_*) versus irradiation time (*t*). *A_0_* and *A_t_* are the absorbance of DPBF at the monitored wavelength before and after irradiation time (*t*). *I_aT_* is the total amount of light absorbed by the dye. *I_aT_* is calculated as a sum of intensities of the absorbed light *Ia* at wavelengths from 530 nm to 800 nm. *I_a_* at given wavelength is calculated using Beer’s law (Equation (2)), where *I_0_* is the transmittance of the filter at a given wavelength and *A* is the absorbance of the dye at this wavelength.

(1) $$\phi_{\Delta }^{S}\;=\;\phi_{\Delta }^{R}\cdot \frac{k^{S}\cdot I_{aT}^{R}}{k^{R}\cdot I_{aT}^{S}}$$

(2) $$I_{a}\;=\;I_{0}\cdot \left(1-e^{-2.3A}\right)\;$$

All spectroscopic measurements for Photophysical Characterization (Section 2.2), were carried out in 1 cm quartz cuvettes (Hellma, Germany) in air-saturated solutions, at room temperature using spectroscopic grade solvents. Absorption spectra were recorded using a double beam UV–Vis–NIR Varian Cary 6000i spectrophotometer (Varian, Palo Alto, CA, USA). Absorption coefficients were derived from the slopes of Lambert-Beer plots. Fluorescence emission spectra were recorded using a Spex Fluoromax-4 spectrofluorometer (Horiba Jobin-Yvon, Edison, NJ, USA). The samples were excited at: **ABAB-1** 667 (THF) and 702 nm (toluene); **A_3_B-1** 665 (THF) and 670 nm (toluene); **A_4_-1** 693 (THF) and 672 nm (toluene). Fluorescent quantum yields (*Φ*_F_) were determined by comparing the integrated fluorescent intensity of optically matched solutions between samples (S) and reference (R) ZnPc in 1-propanol (*Φ*_F_ = 0.20) [22]. The samples were excited at: **ABAB-1** 609 (THF) and 603 nm (toluene); **A_3_B-1** 610 (THF) and 605 nm (toluene); **A_4_-1** 610.5 (THF) and 610 nm (toluene). Fluorescence intensity was corrected using the refractive index of the solvents used (Equation (3)).

(3) $$\Phi_{F}\;=\;\frac{Area_{S}\cdot n^{2}{}_{S}}{Area_{R}\cdot n^{2}{}_{R}}$$

Time-resolved fluorescence decays were measured using a Fluotime 200 time-correlated fluorescence lifetime spectrophotometer (PicoQuant GmbH, Berlin, Germany), equipped with a red sensitive photomultiplier. Excitation was achieved by means of a 654 nm picosecond laser working at 1 MHz repetition rate. The counting frequency was always below 1%. Fluorescence lifetimes were analyzed using PicoQuant FluoFit 4.0 data analysis software.

^1^O_2_ generation in THF and toluene was studied by time-resolved near-infrared phosphorescence by means of a customized setup [33,34]. Briefly, a pulsed Nd:YAG laser (FTSS355-Q, Crystal Laser, Berlin, Germany) working at 1 kHz repetition rate (for THF and toluene) at 355 nm (third harmonic, 0.5 μJ per pulse) was used to excite the sample. A 1064-nm rugate notch filter (Edmund Optics) and an uncoated SKG-5 filter (CVI Laser Corporation) were placed in the laser path to remove any residual NIR emission. The light emitted by the sample was filtered with a 1100 nm long-pass filter (Edmund Optics) and later by a narrow bandpass filter at 1275 nm (BK-1270-70-B, bk Interferenzoptik). A thermoelectric-cooled NIR-sensitive photomultiplier tube assembly (H9170-45, Hamamatsu Photonics, Hamamatsu, Japan) was used as detector. Photon counting was achieved with a multichannel scaler (NanoHarp 250, PicoQuant, Berlin, Germany). The time dependence of the ^1^O_2_ phosphorescence with the signal intensity S(*t*) is described by Equation (4), in which *τ_T_* and *τ*_Δ_ are the lifetimes of the photosensitizer triplet state and of ^1^O_2_ respectively, and S(*0*) is a pre-exponential parameter proportional to *Φ*_Δ_. Curve fitting was performed with the Graphpad Prism version 7.00 for Windows, (GraphPad Software, La Jolla California USA, www.graphpad.com). *Φ*_Δ_ was determined by comparing the S(*0*) values of optically matched solutions of the drug and the reference (phenalenone (PN), in toluene and THF *Φ*_Δ_ = 0.99 and 0.96 respectively) [35,36], at 355 nm as described by Equation (5).

(4) S(t) =S(0)·τΔτΔ−τT·(e−tτΔ−e−tτT)

(5) $$\Phi_{\Delta }^{S}\;=\;\Phi_{\Delta }^{R}\;\cdot \frac{S{\left(0\right)}_{S}}{S{\left(0\right)}_{R}}\;$$

### 3.4. Determination of the Log Po/w

Initially, equal volumes of *n-*octanol and water were mixed vigorously for 3 days at 25 °C to promote solvent saturation in both phases. Each sample of Zn(II)Pc **1** was then added from stock solutions in DMSO to 2 mL of the mixture (%DMSO < 1%, [Zn(II)Pc] = 10^-5^ M) and stirred for 30 min; next, they were incubated 1 h at room temperature. After separation, 10 μL of *n-*octanol phase and 10 μL of PBS phase were taken and diluted by DMSO to 1.01 mL. The UV–Vis spectra of both phases were recorded, and the partition coefficient was calculated based on the absorbance values at *Q-*band maxima (702 nm for **ABAB-1**, 685 nm for **A_3_B-1** and 681 nm for **A_4_-1**). The results are the average of three independent measurements.

$$logP_{OW}\;=\;log\left(\frac{A{\left(DMSO\right)}_{O}\cdot V_{W}}{A{\left(DMSO\right)}_{W}\cdot V_{O}}\right)$$

### 3.5. Biological Assays

#### 3.5.1. Cell Culture

HeLa (cervical adenocarcinoma cells) and SCC-13 (skin squamous carcinoma cells) human cancer cell lines were used for cell cultures. They were grown using DMEM (Dulbecco’s Modified Eagle’s medium) culture media, supplemented with 10% Foetal Bovine Serum (FBS) and 1% antibiotics (G penicillin, 100 U/mL; streptomycin, 100 µg/mL), purchased from Fisher. They were incubated in HERACell incubator (Heraus) at 37 °C with 5% CO_2_ concentration and 95% relativity humidity.

#### 3.5.2. Photodynamic Treatment

Solutions of **ABAB-1**, **A_3_B-1**, and **A_4_**-**1** were prepared in concentrations of 1·10^−6^ and 1·10^−7^ M and added to cell cultures in well plates P24 with DMEM (supplemented with antibiotics but without FBS). The cells were incubated with each Pc (previously dissolved in DMSO keeping the DMSO content below 1%) for 5 h in darkness, immediately were irradiated with different red light doses (3, 6 and 9 J/cm^2^). Light source was constituted by a rectangular matrix with 384 LED (light emitting diodes), (WP7143 SURC, Kingbright) with an emission peak of 637 nm and a bandwidth of ± 17 nm. The fluence rate used was 12.7 mW/cm^2^. After irradiation, cell culture was replaced by fresh media supplemented with antibiotics and FBS, and cells were incubated 24 h until their evaluation.

#### 3.5.3. Cell Viability

Cell survival was determined after photodynamic treatment, both darkness plates and irradiated plates, through MTT assays. This is a colorimetric technique based on mitochondrial enzymes capacity of alive cells to reduce the water-soluble compound 3-(4,5-dimethylthiazol-2-yl)-2,5-diphenyltetrazolium bromide (MTT) to formazan, an insoluble compound which has a purple colour. After 24 h of incubation, MTT was added to well plates in a final concentration of 100 µg/mL, for 3 h at 37 °C. Then, DMSO (Panreac) was added in order to solve formazan and optical density was measured in SpectraFluor plate reader (Tecan), with a wavelength of 542 nm.

#### 3.5.4. Measurement of Intracellular ROS

The intracellular production of ROS in SCC-13 and HeLa cells was evaluated as previously described [37]. Cells were incubated with Zn(II)Pcs (1·10^−6^ M) for 5 h, and during the last hour 2,7-dichloro-dihydrofluorescein diacetate (DHF-DA, Abcam) was added to the cultures, reaching a final concentration of 6·10^−6^ M. Afterwards, and without removing DHF-DA, cells were exposed to green light (3 and 6 J/cm^2^) and immediately after irradiation analyzed by fluorescence microscopy, under blue excitation light (λexc = 436 nm). ROS production was quantified by using Image J after measuring green fluorescence.

#### 3.5.5. Optical Microscopy and Statistical Analysis

Microscopic observations were carried out using an Olympus BX61 epifluorescence microscope equipped with green filter (exciting filter BP510-550). Photographs were obtained with the digital camera Olympus CCD DP70 and processed using the Adobe Photoshop CS5 extended version 12.0 software (Adobe Systems Inc., San Jose, CA, USA).

Data are expressed as the mean value of at least three experiments ± standard deviations (SD). The statistical significance was determined using analysis of variance (ANOVA) followed by Bonferroni’s test, and *p* < 0.05 was considered statistically significant.

#### 3.5.6. Subcellular Localization

In order to determine intracellular localization of Zn(II)Pcs, cells were grown in coverslips in P12 well plates and they were incubated with the PS in a concentration of 10^−6^ M for 18 h at 37 °C. After incubation, cells were washed with PBS and were observed with fluorescence microscopy.

## 4. Conclusions and Future Perspectives

Three amphiphilic polyethyleneglycol-functionalized Zn(II)Pcs, namely, **ABAB-1**, **A_3_B-1**, and **A_4_-1**, have been synthesized in the search for new PS for PDT applications. **ABAB-1**, **A_3_B-1** are both obtained in a crossed-condensation reaction of two differently substituted phthalonitriles, combining bulky fluorinated substituents in the **B** isoindoles, which have previously proved effective in suppressing aggregation and increasing the ^1^O_2_ generation efficiency, and hydrophilic triethyleneglycol substituents in **A** isoindoles to impart water solubility. On the other hand, **A_4_-1** is an octa(triethyleneglycol) Pc which has been prepared to experimentally demonstrate that the presence of the bulky fluorinated substituents is fundamental to achieve good photosensitization abilities. In fact, photophisycal and aggregation studies have determined that **ABAB-1** is the compound that most efficiently generates ^1^O_2_ (*τ*_Δ_ trend is **ABAB-1 > A_3_B-1** > **A_4_**-**1),** and is less prone to aggregate in water media. Finally, in vitro assays have been carried out in order to determine the toxicity for tumor cells for the three Zn(II)Pcs. It has been proved that none of these Pcs are toxic for tumor cells in darkness, but when the tissue is irradiated in the presence of the PS, fatality turns out. Moreover, consistently with the ^1^O_2_ generation studies, the impact is higher in the case of **ABAB-1** and **A_3_B-1**, than **A_4_-1**. These findings make Zn(II)Pcs with bulky bis(trifluoromethyl)phenyl groups, particularly those with an ABAB pattern candidates of choice as PS. The next step in this research is to incorporate groups with extended hydrophilicity, and also bind molecules for specific targeting that would allow increasing selectivity against different tumor tissues.

## Figures and Tables

**Table 1:** Photophysical properties of Zn(II)Pcs **ABAB-1**, **A_3_B-1** and **A_4_-1** in toluene and THF.

Sample	Solvent	Log*ε* (*λ*/nm)	*λ_f_*/nm	*Φ*_F_	*τ_S_*/ns	*τ_T_*/μs	*Φ*_Δ_
**ABAB-1**	toluene	4.73 (328), 5.09 (674), 5.12 (712) ^a^	715	0.13	1.7	0.35 ^b^	0.77
**A_3_B-1**	toluene	4.63 (349), 4.97 (679) ^a^, 4.94 (697)	702	0.17	2.1	0.47 ^b^	0.71
**A_4_-1**	toluene	4.50 (349), 5.03 (682) ^a^	684	0.43	3.0	0.45 ^b^	0.61
**ABAB-1**	THF	4.82 (349), 5.08 (671), 5.09 (703) ^a^	709	0.13	1.7	0.30 ^c^	0.83
**A_3_B-1**	THF	4.71 (348), 5.03 (675) ^a^	692	0.28	2.2	0.23 ^c^	0.74
**A_4_-1**	THF	4.61 (349), 5.12 (676) ^a^	682	0.25	3.0	0.37 ^c^	0.68

^a^ λ absorption maxima; ^b^ uncertainty of ± 0.05; ^c^ uncertainty of ± 0.08.

**Table 2:** Toxicity effects in SCC-13 and HeLa cells induced by red light, in the absence of any Pcs, and by incubation with either of the Zn(II)Pcs **1** in the dark. Cell toxicity was evaluated by the MTT assay, 24 h after treatment.

Compound	Concentration [M]	Surviving Fraction (% ± SD) ^1^	
		SCC-13	HeLa
**No Zn(II)Pc – Control dark**		101 ± 2.4	100 ± 1.1
No Zn(II)Pc – Control dark + 1% DMSO		102 ± 4.3	98 ± 3.7
No Zn(II)Pc – Control light (9 J/cm^2^)		100 ± 1.7	99 ± 2.3
No Zn(II)Pc – Control light (9 J/cm^2^) + 1% DMSO		101 ± 2.8	101 ± 1.5
**ABAB-1**	1 × 10^−6^	98 ± 5.4	101 ± 5.5
	1 × 10^−7^	102 ± 3.1	95 ± 2.8
**A_3_B-1**	1 × 10^−6^	97 ± 6.3	97 ± 3.5
	1 × 10^−7^	99 ± 5.5	99 ± 3.6
**A_4_-1**	1 × 10^−6^	99 ± 4.5	97 ± 5.5
	1 × 10^−7^	98 ± 5.0	98 ± 2.8

^1^ Data are expressed as mean values obtained from three independent experiments ± standard deviation (SD).

## Supplementary Materials

The following are available online.

## Appendix A

**Materials and methods for subcellular localization of Figure A1:** To analyze the intracellular localization of organelles, SCC-13 and HeLa cells were grown on coverslips and, incubated with known fluorescent probes for lysosomes (LysoTracker Green DND-26, Invitrogen), mitochondria (MitoTracker Green FM, Invitrogen), or Golgi apparatus (NBD, C6-ceramide (*N-*[6-[(7-nitro-2-1,3-benzoxadiazol-4-yl)amino]hexanoyl]-*D-*erythro-sphingosine, Invitrogen)) at the concentrations indicated by the suppliers. Then, cells were briefly washed in PBS, mounted on slides with a drop of PBS and immediately observed under the fluorescence microscope.
