# Supplementary material for: Assessing Amphiphilic ABAB Zn(II) Phthalocyanines with Enhanced Photosensitization Abilities in In Vitro Photodynamic Therapy Studies Against Cancer
Source: Molecules. 2020 Jan 4;25(1):213. doi: 10.3390/molecules25010213 (PMC6983099; doi:10.3390/molecules25010213)

Assessing amphiphilic ABAB Zn(II) phthalocyanines with enhanced photosensitization abilities in *in vitro* photodynamic therapy studies against cancer

Miguel Á. Revuelta-Maza [1], Marta Mascaraque [1,4], Patricia González-Jiménez [1], Arturo González-Camuñas [1], Santi Nonell [3], Ángeles Juarranz [1,4*], Gema de la Torre [1*] and Tomás Torres [1,2*]

[1] Departments of Organic Chemistry and Biology, Universidad Autónoma de Madrid. C/Francisco Tomás y Valiente 7, Madrid 28049, Spain
[2] Instituto Madrileño de Estudios Avanzados (IMDEA)-Nanociencia. C/Faraday 9, Cantoblanco, Madrid 28049, Spain
[3] Institut Químic de Sarrià. Universitat Ramon Llull, Barcelona 08017, Spain
[4] Instituto Ramón y Cajal de Investigación Sanitaria, Madrid 28034, Spain

* Correspondence: tomas.torres@uam.es (T.T.); gema.delatorre@uam.es (G.T.); angeles.juarranz@uam.es (A. J,) Tel.: +34-91-4974151

SUPPORTING INFORMATION

1. RELEVANT FIGURES MENTIONED IN THE TEXT

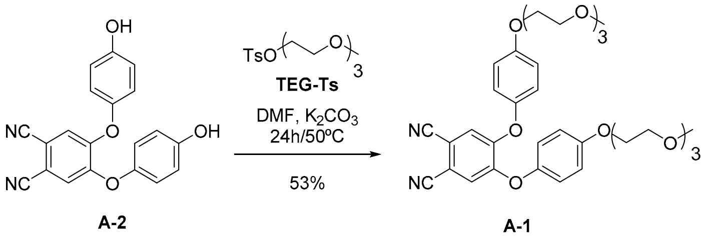

*Scheme S 1. Synthesis of ftalonitrile **A-1** from **A-2**.*

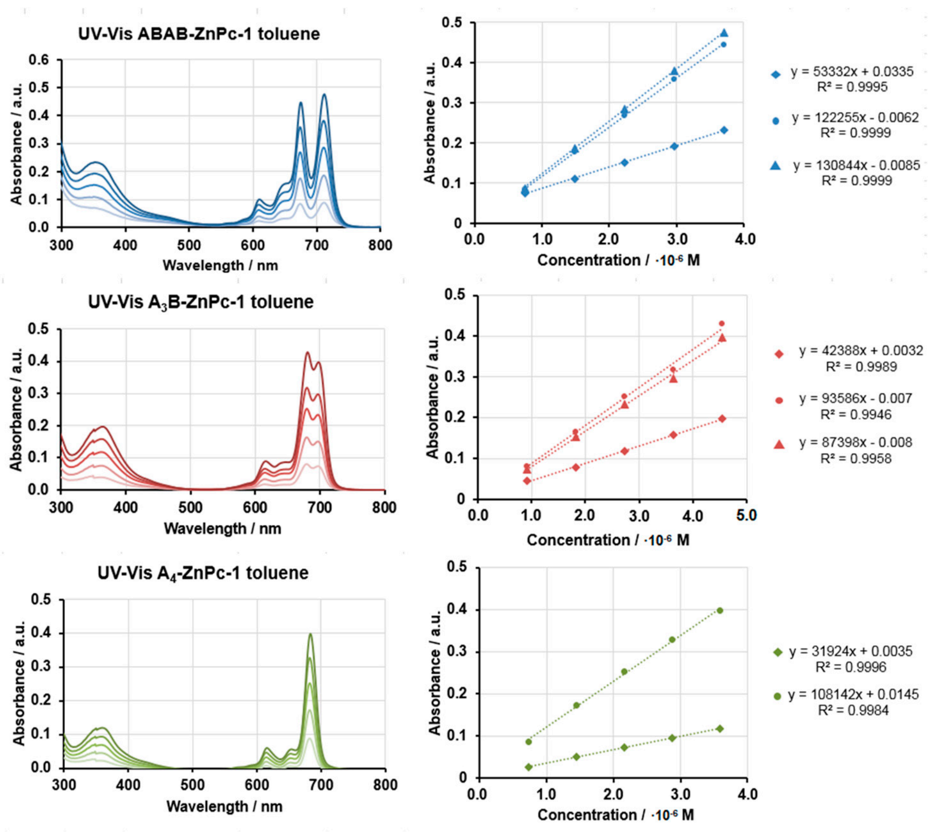

*Figure S1. UV-vis spectra for concentration-dependent studies of **ABAB-1**, **A_3B-1** and **A_4-1** in toluene. Linear regression between maxima intensity and concentration.*

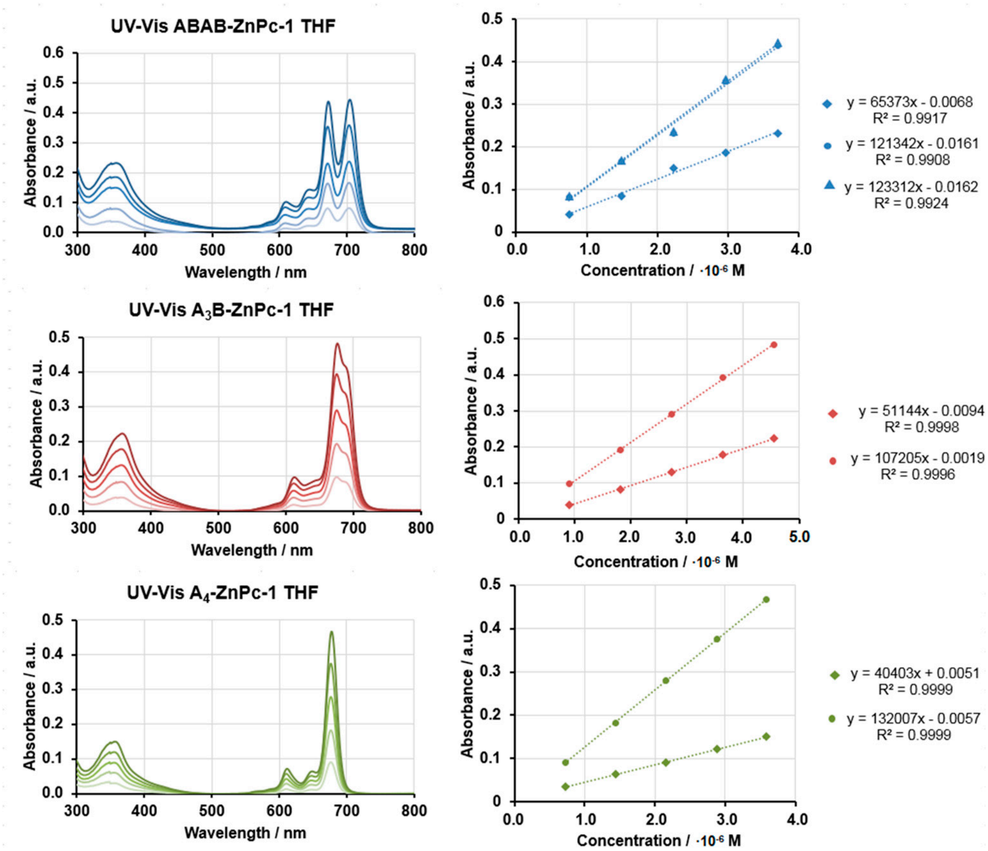

Figure S2. UV-vis spectra for concentration-dependent studies of ***ABAB-1***, ***A3B-1*** *and* ***A4-1*** *in THF. Linear regression between maxima intensity and concentration.*

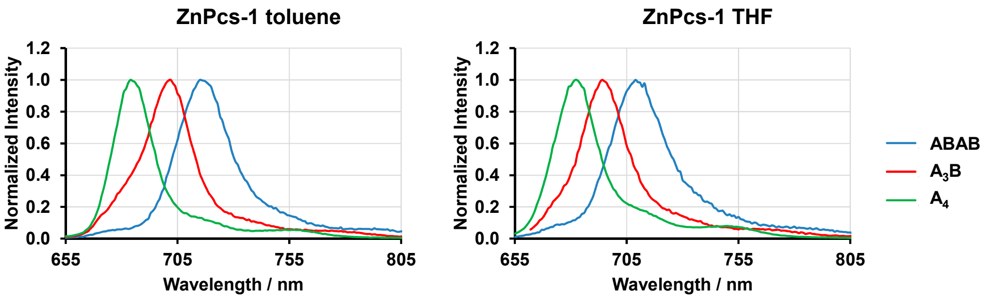

Figure S3. Normalized fluorescence spectra of ***ABAB-1, A3B-1*** *and* ***A4-1*** *in toluene and THF solutions*

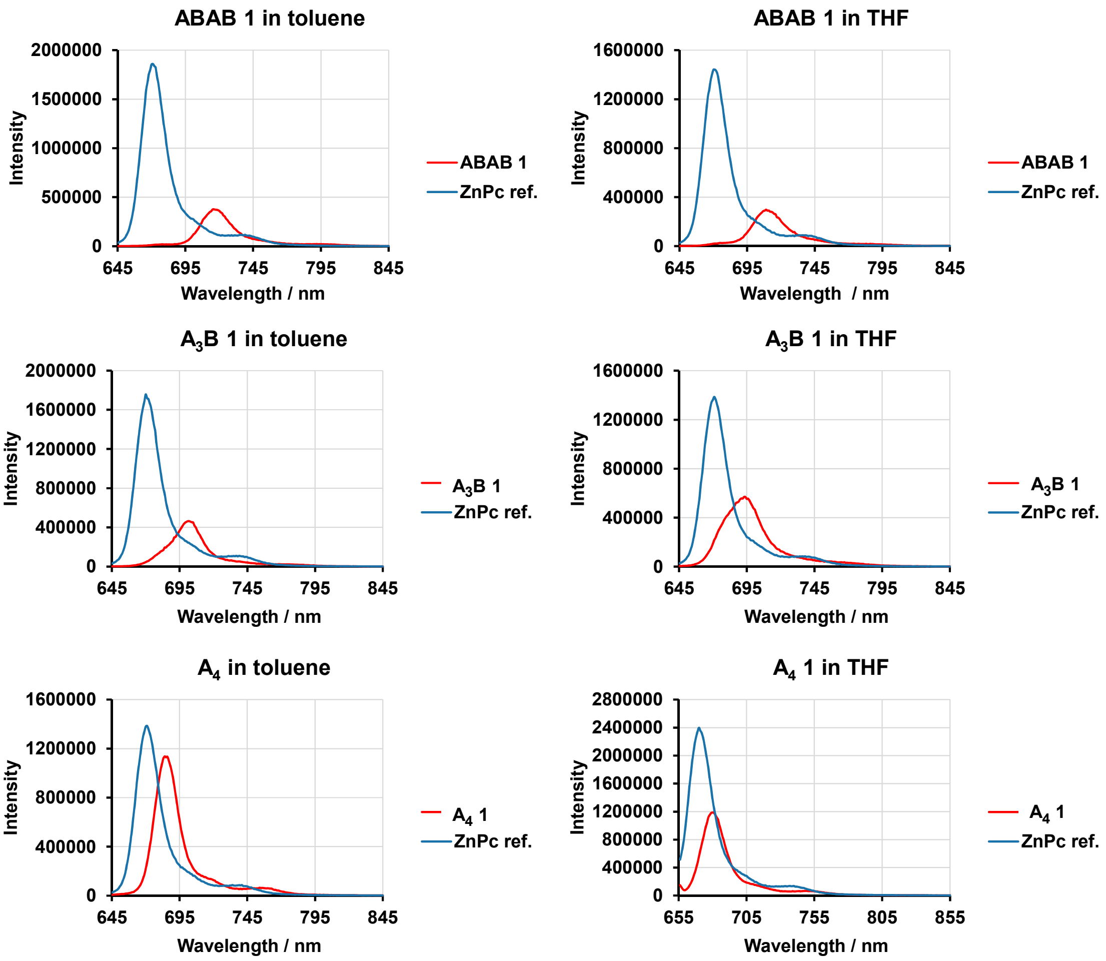

*Figure S4. Calculation of fluorescence quantum yields in toluene and THF solutions of **ABAB-1**, **A_3B-1** and **A_4-1**.*

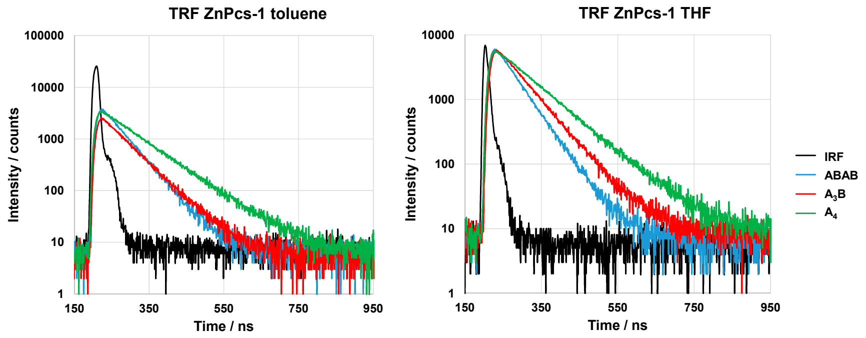

*Figure S5. Time-resolved fluorescence spectra of **ABAB-1**, **A_3B-1** and **A_4-1** in toluene and THF.*

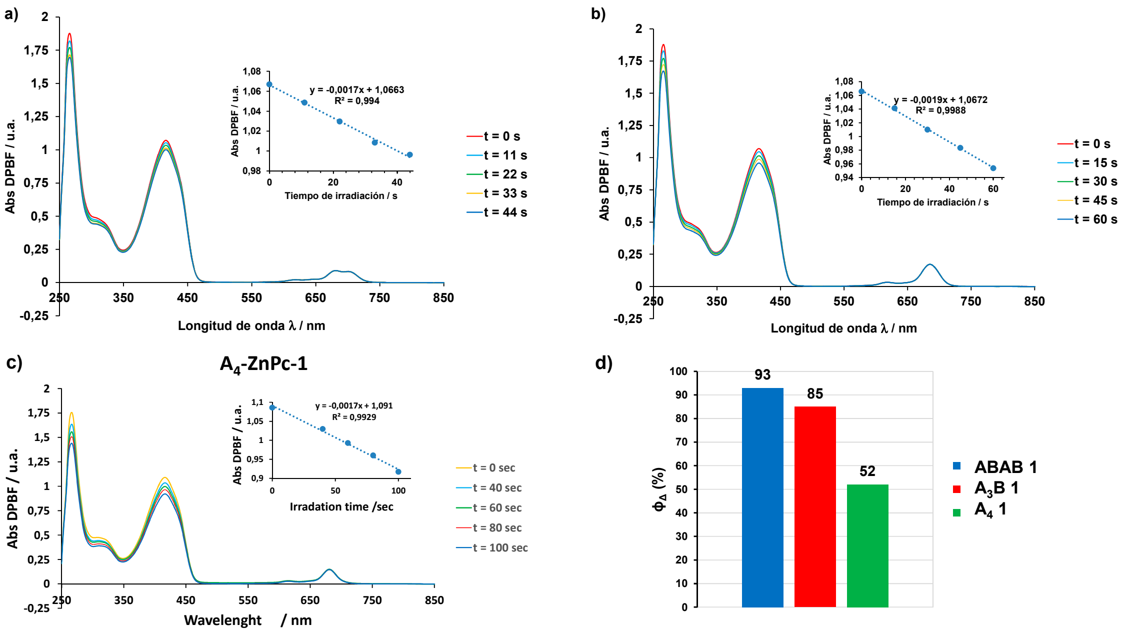

*Figure S6. a-c) Absorbance decrease of DPBF over time due to $^1O_2$ photoinduced generation by **ABAB-1, A$_3$B-1** and **A$_4$-1** in DMSO. d) Representation of the relative $^1O_2$ efficiency of **ABAB-1** (93%), **A$_3$B-1** (85%) and **A$_4$-1** (52%).*

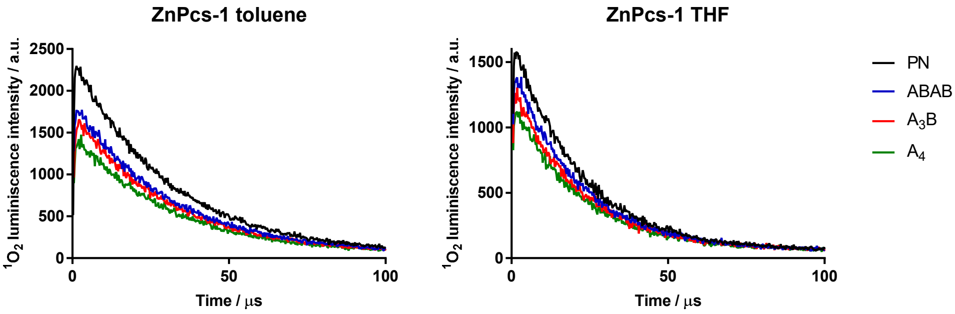

*Figure S7. $^1O_2$ production of **ABAB-1, A$_3$B-1** and **A$_4$-1 1** with regard to the reference phenalenone (PN) in toluene and THF.*

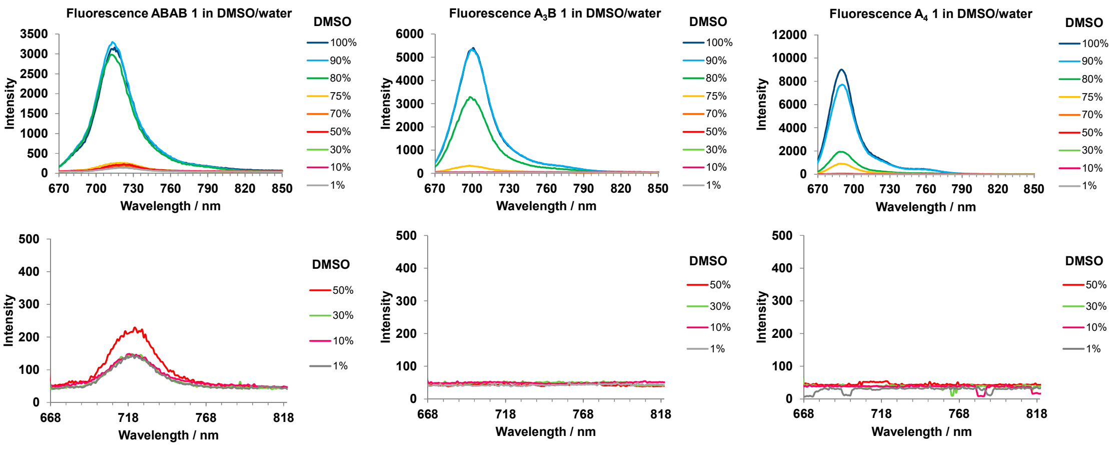

*Figure S8. Fluorescence spectra of **ABAB-1, A$_3$B-1** and **A$_4$-1** in different DMSO/water ratios.*

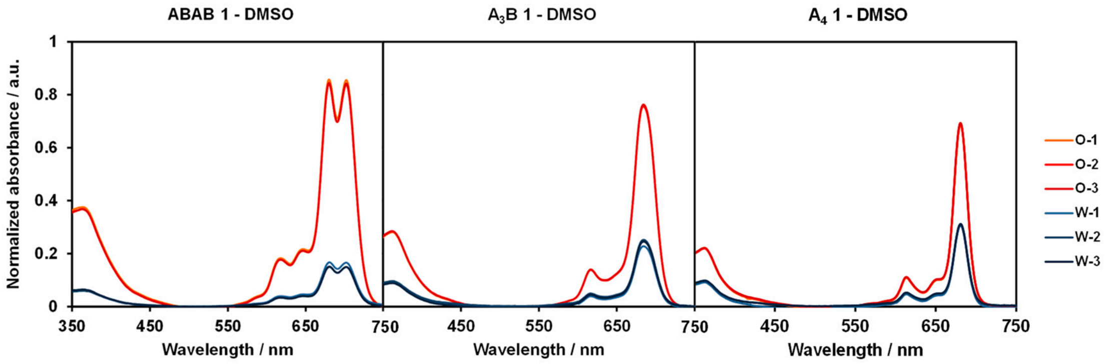

*Figure S9. UV-vis spectra of the octanol (O) and water (W) phases (three consecutive experiments) in n-octanol/water partition experiments with **ABAB-1**, **A_3B-1** and **A_4-1**.*

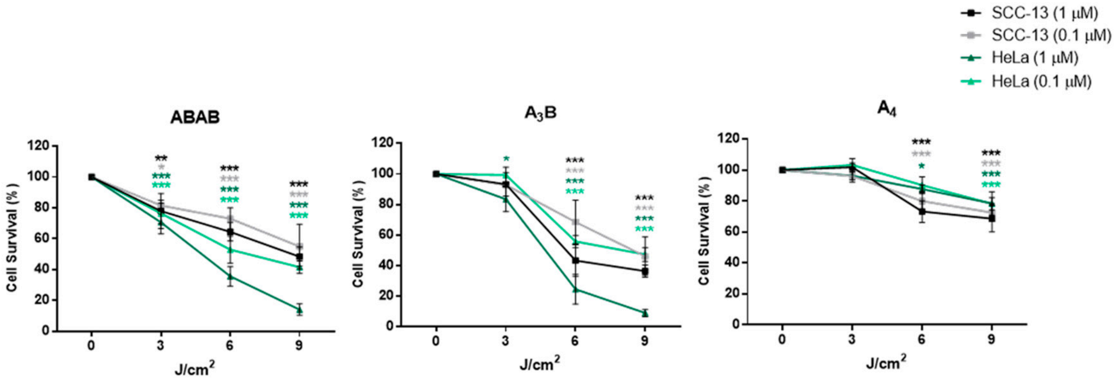

*Figure S10. Phototoxicity induced by **ABAB-1, A3B-1** and **A4-1** in SCC-13 and HeLa cells, which were incubated with concentrations of 1·10-6 or 1·10-7 M for 5 h and then irradiated with red light at variable doses. The response was dependent, on both cells line, of the concentration of Pcs and the light dose. Each value corresponds to the mean obtained from three independent experiments ± SD. (* $p < 0.05$; ** $p<0.01$; *** $p < 0.001$).*

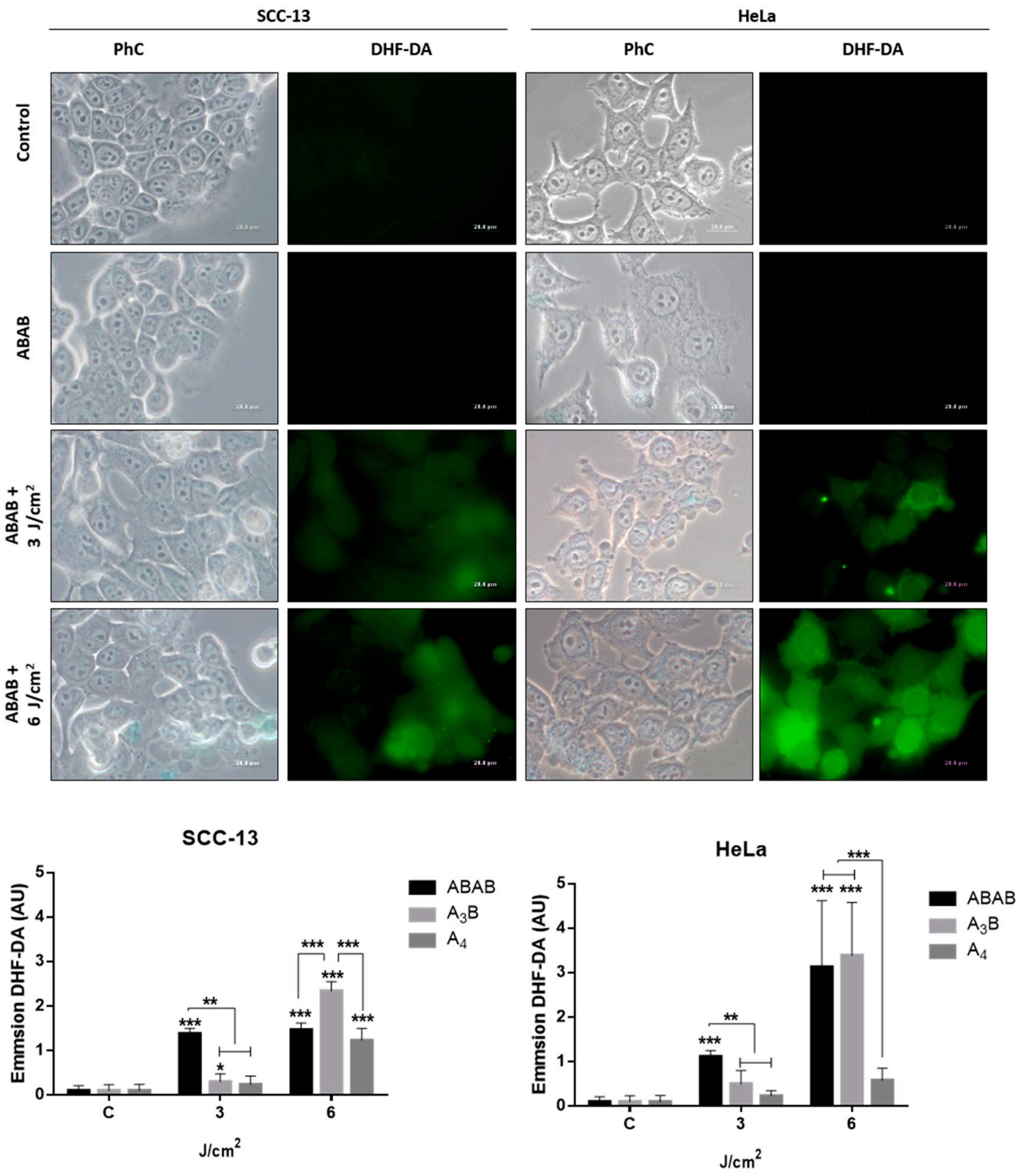

*Figure S11. ROS production detected by the DHF-DA fluorescent probe after PDT with Zn(II)Pcs **ABAB-1, A_3B-1** and **A_4-1** and red light. Cells were incubated with 1·10-6 M Pcs for 5 h and in the last hour of incubation DHF-DA was added, to a final concentration of 6 μM. Cells were exposed to red light (3 and 6 J/cm2). The fluorescence signal was observed by fluorescence microscopy (λexc = 436 nm). Representative photographs of cells subjected to ABAB-PDT. Intracellular fluorescence intensity of all Zn(II)Pcs was measured by ImageJ . ***P<0.001.*

2. NMR AND MASS SPECTROMETRY CHARACTERIZATION

4,5-bis-(4-(2-(2-(2-methoxyethoxy)ethoxy)ethoxy)phenoxy)phthalonitrile (A-1):

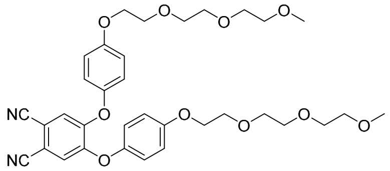

MS and HR-MS:

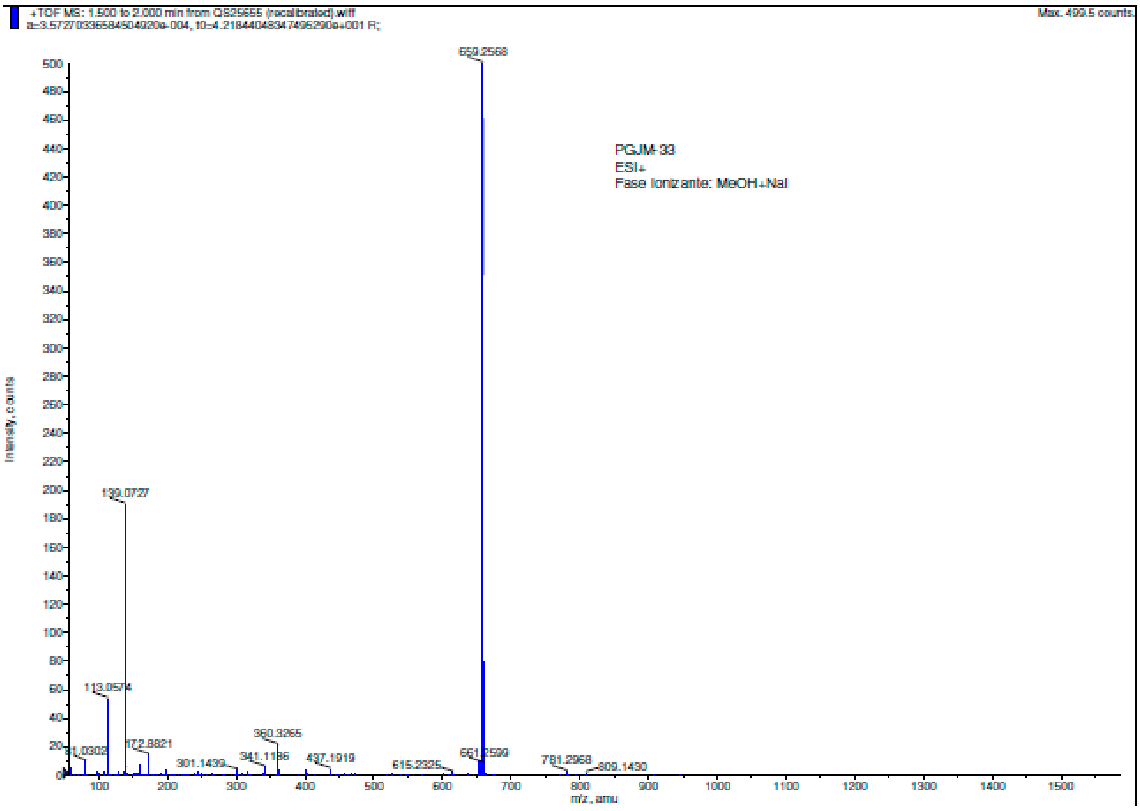

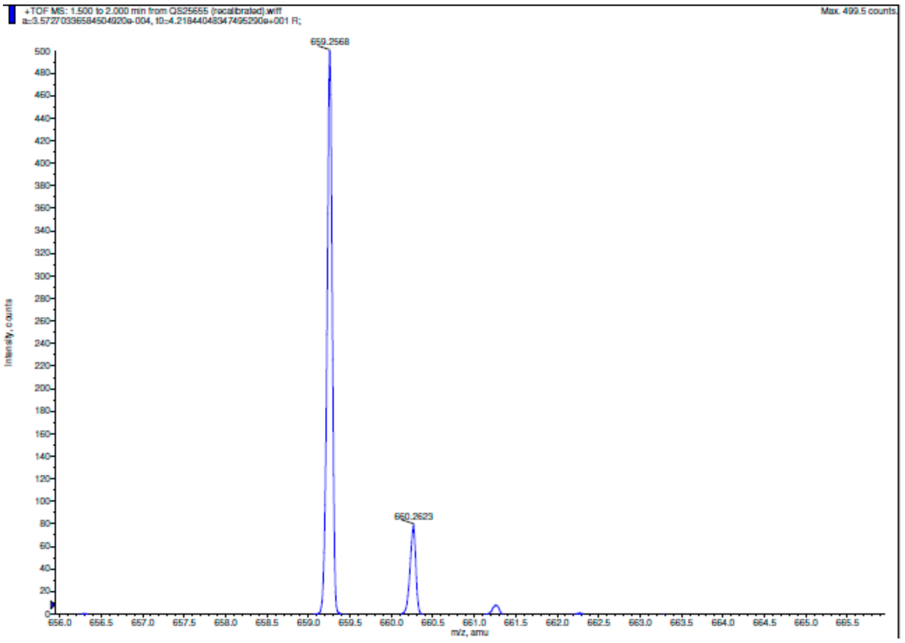

+TOF MS: 1.500 to 2.000 min from QS25655 (recalibrated).wiff
a=3.57270336584504920e-004, t0=4.21844048347495290e+001 R;
Max. 499.5 counts.
659.2568
660.2623
Intensity, counts
m/z, amu

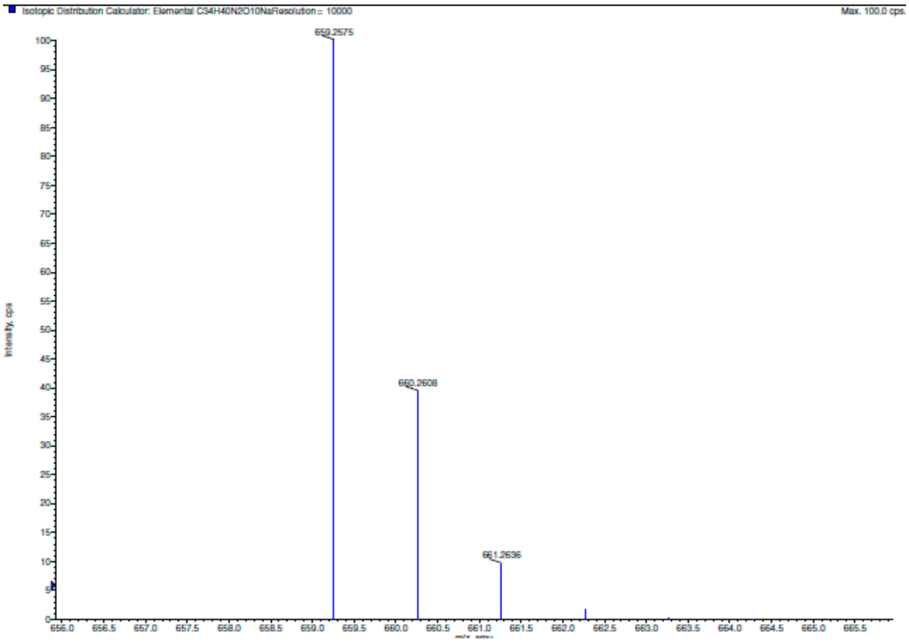

Isotopic Distribution Calculator: Elemental C34H40N2O10NaResolution = 10000
Max. 100.0 cps.
659.2575
660.2608
661.2636
Intensity, cps

^{1}H NMR in $CDCl_3$:

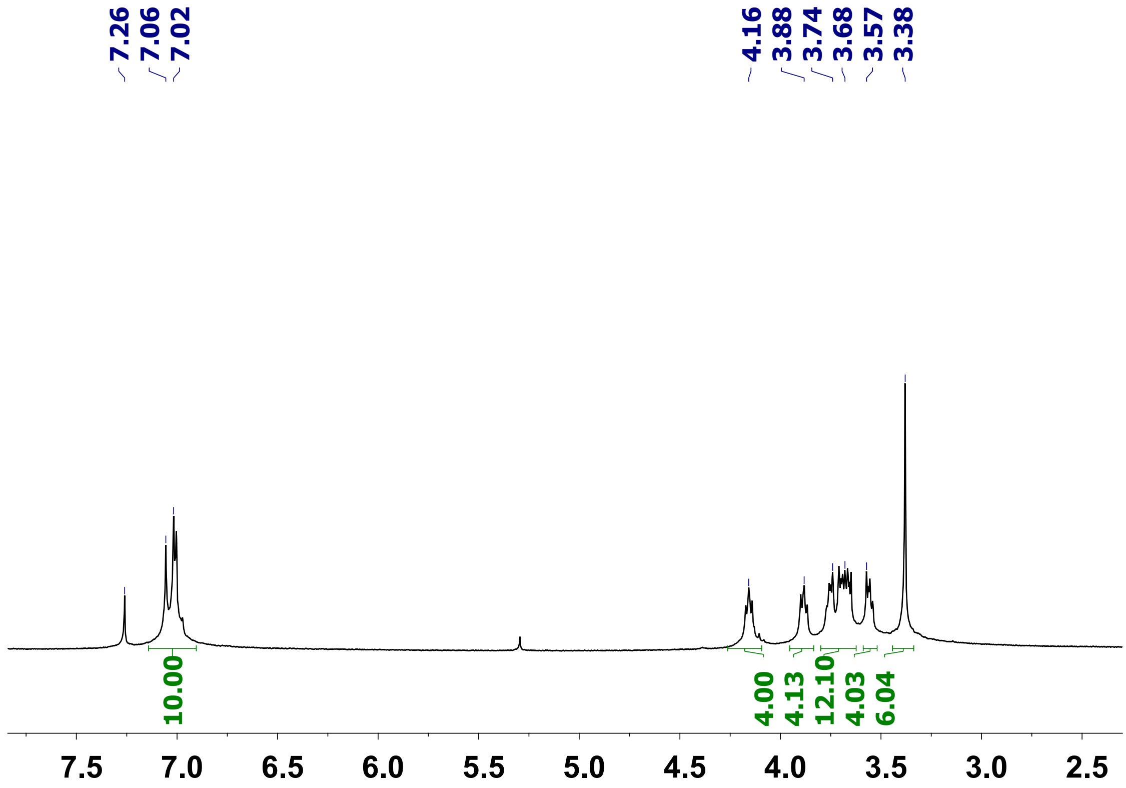

^{13}C NMR in $CDCl_3$:

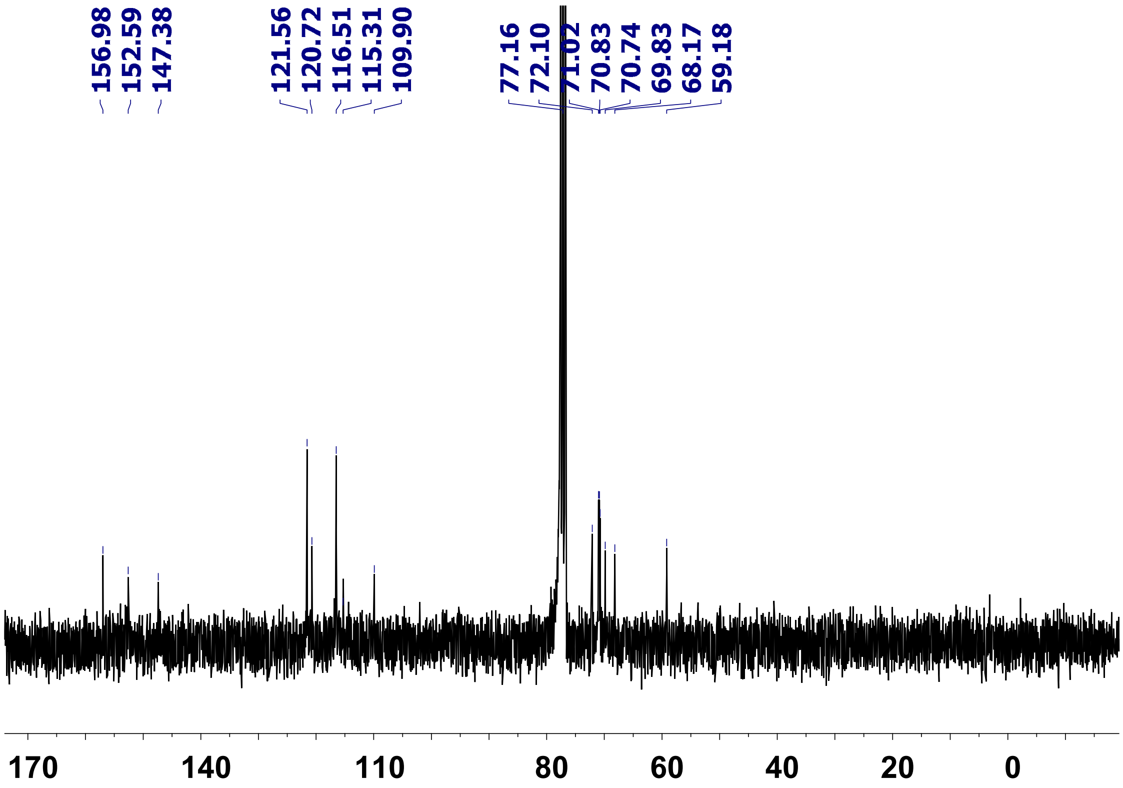

A$_3$B ZnPc 2:

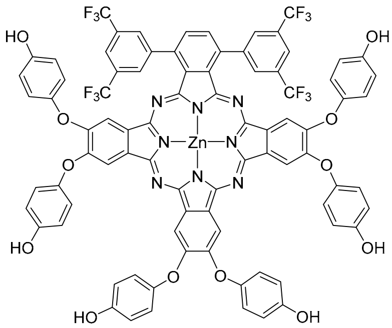

MS and HR-MS:

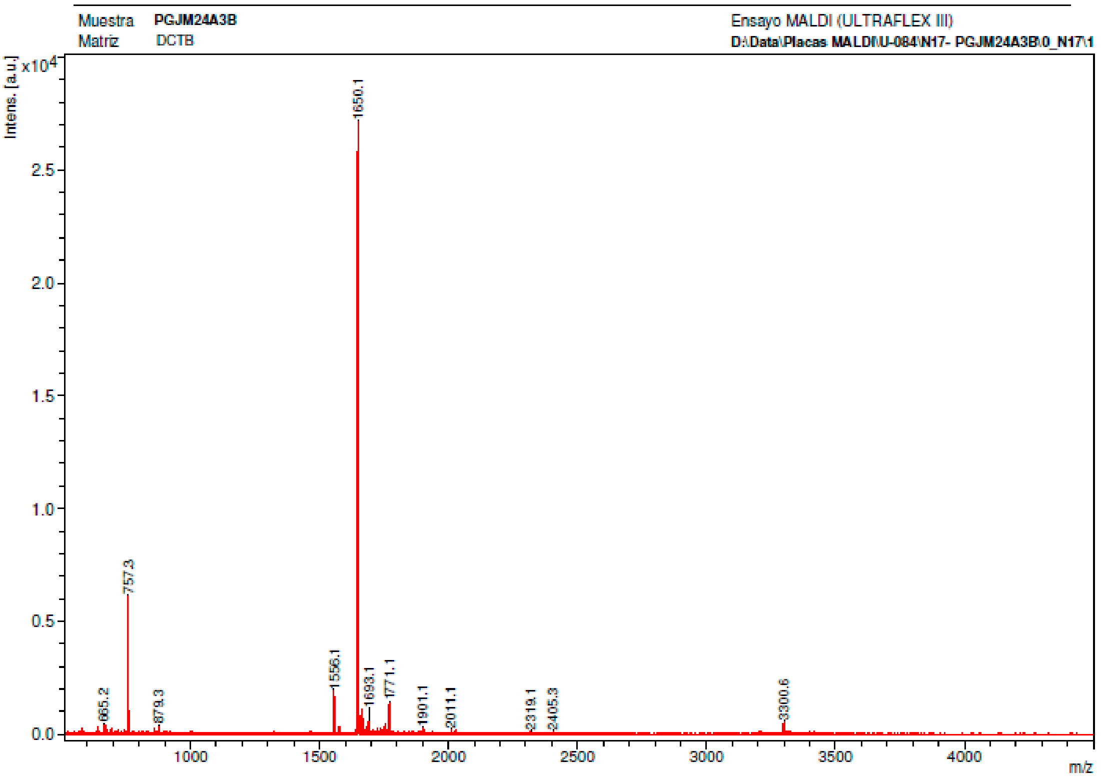

Muestra PGJM24A3B
Matriz DCTB
Ensayo MALDI (ULTRAFLEX III)
D:\Data\Placas MALDI\U-084\N17- PGJM24A3B\0_N17\1
Intens. [a.u.]
x10^4
1650.1
757.3
665.2
879.3
1556.1
1693.1
1771.1
1901.1
2011.1
2319.1
2405.3
3300.6
m/z

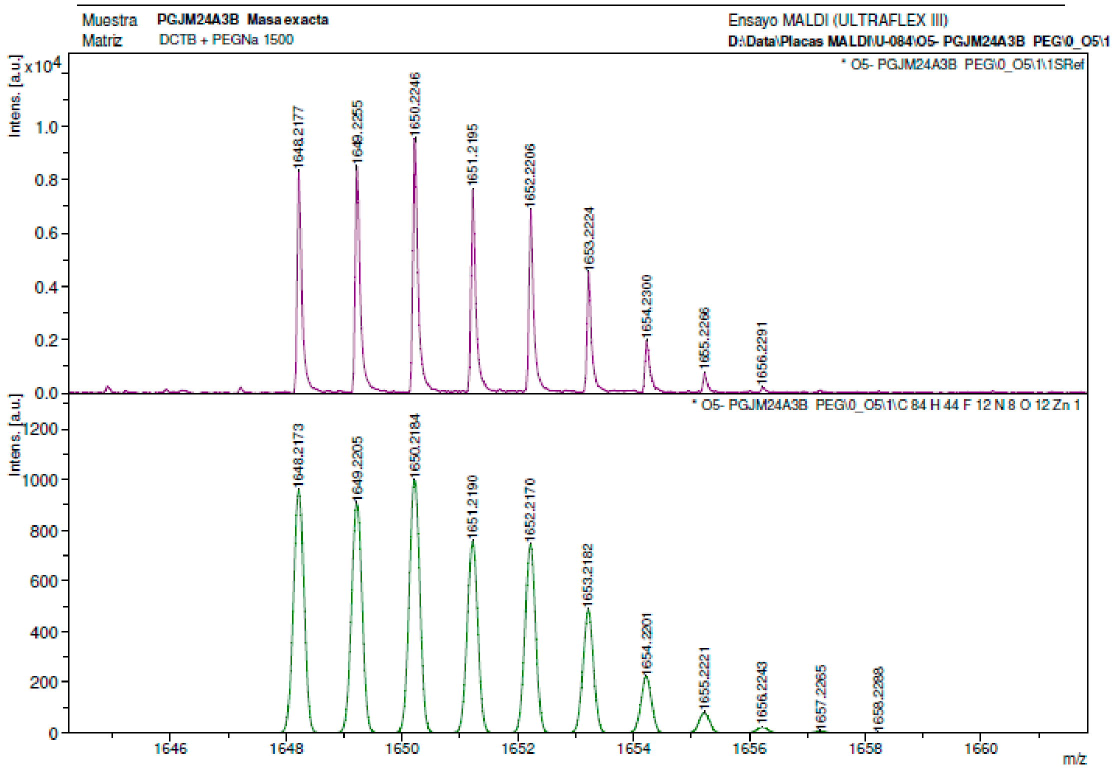

Muestra PGJM24A3B Masa exacta
Matriz DCTB + PEGNa 1500
Ensayo MALDI (ULTRAFLEX III)
D:\Data\Placas MALDI\U-084\O5- PGJM24A3B PEG\0_O5\1
* O5- PGJM24A3B PEG\0_O5\1\1SRef
Intens. [a.u.]
x10^4
1648.2177
1649.2255
1650.2246
1651.2195
1652.2206
1653.2224
1654.2300
1655.2266
1656.2291
* O5- PGJM24A3B PEG\0_O5\1\C 84 H 44 F 12 N 8 O 12 Zn 1
1648.2173
1649.2205
1650.2184
1651.2190
1652.2170
1653.2182
1654.2201
1655.2221
1656.2243
1657.2265
1658.2288
m/z

^{1}H NMR in DMSO-d6:

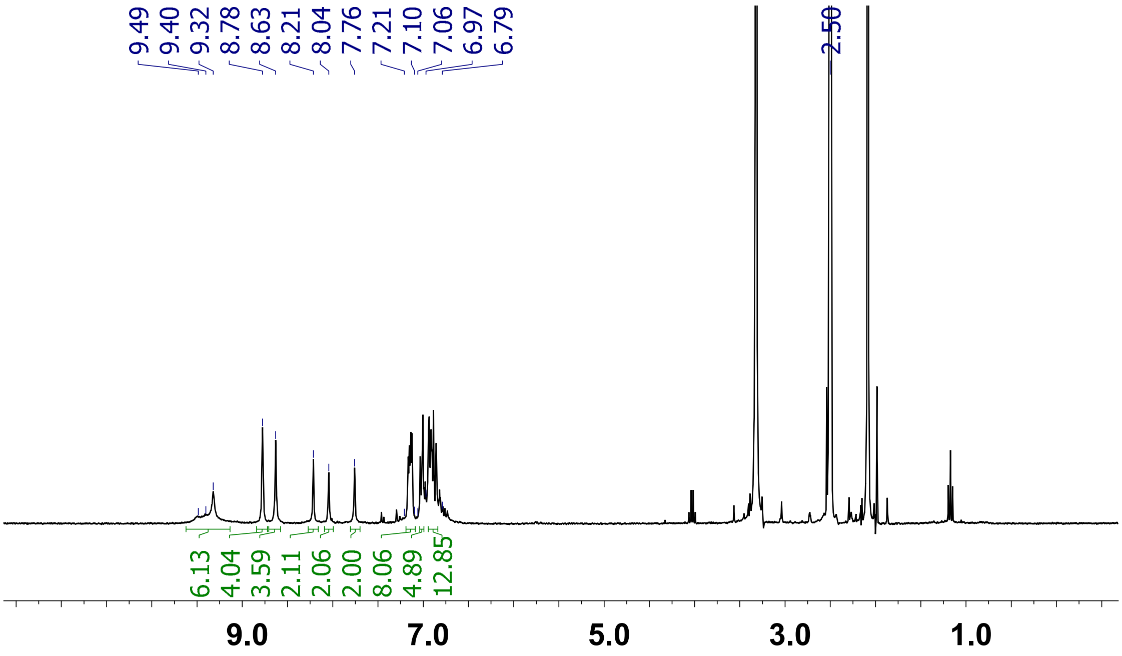

^{13}C NMR in THF-d8:

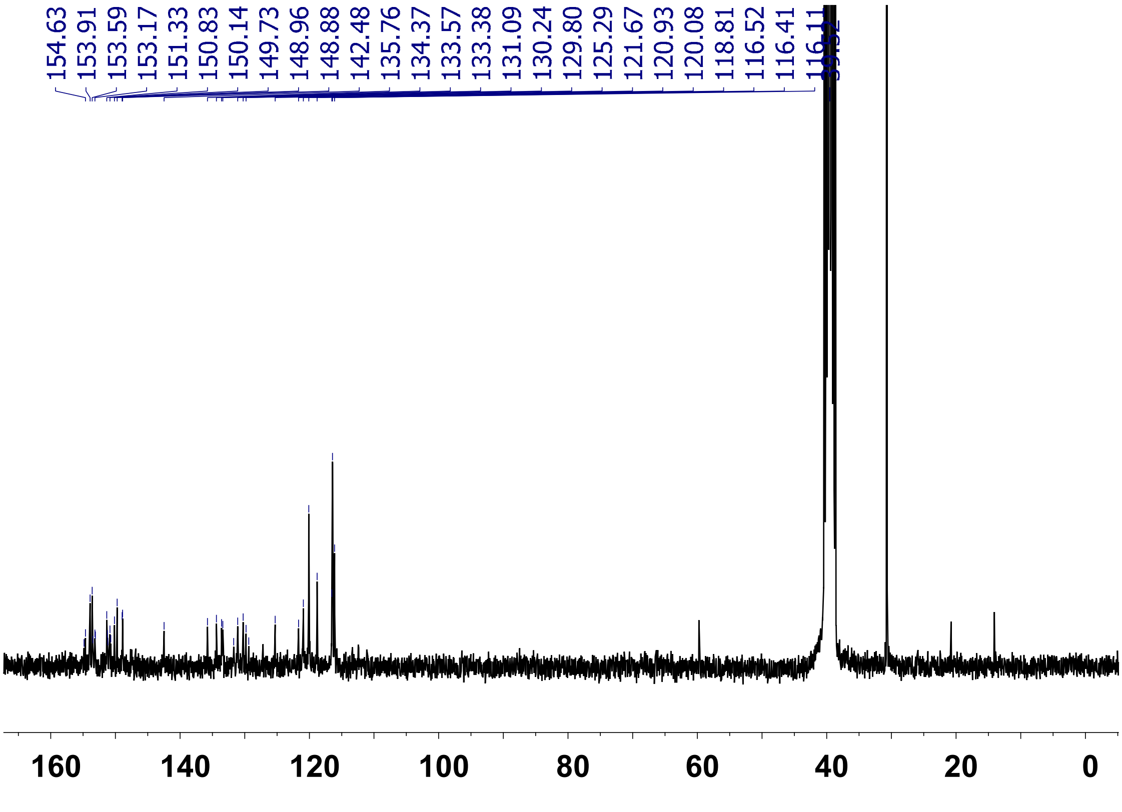

ABAB Zn(II)Pc 1:

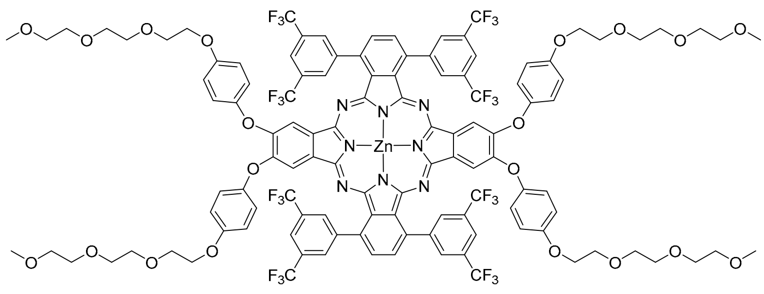

MS and HR-MS:

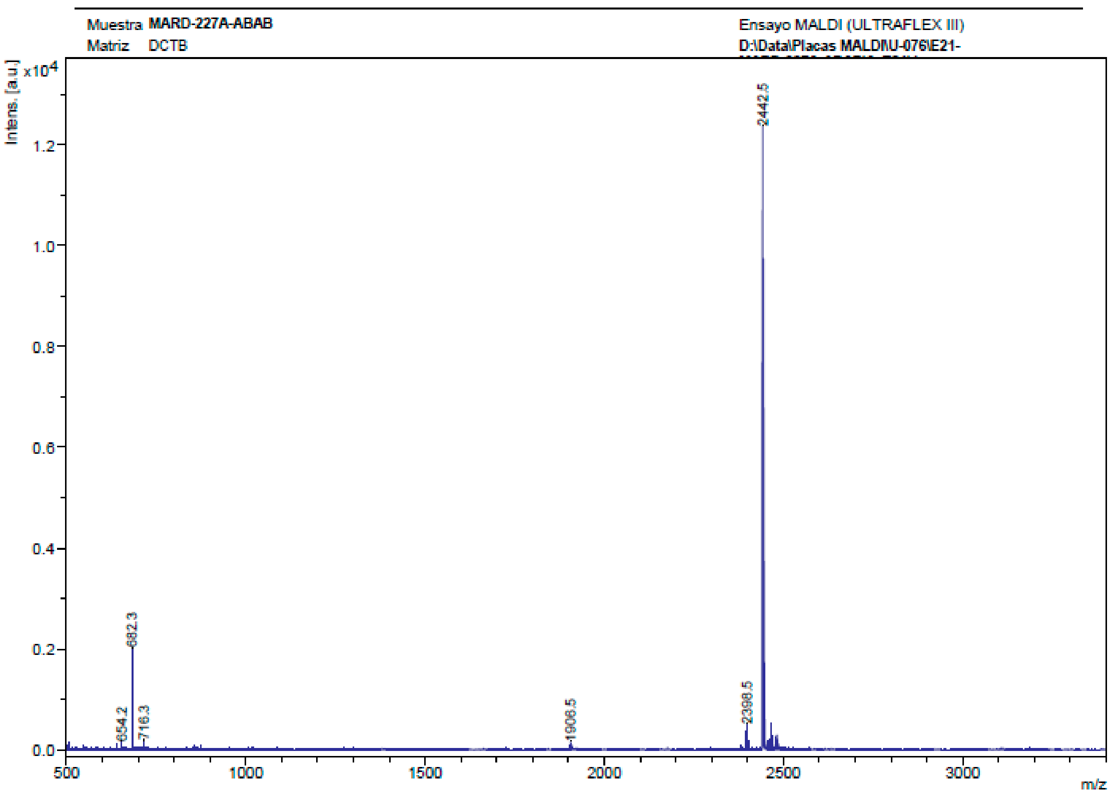

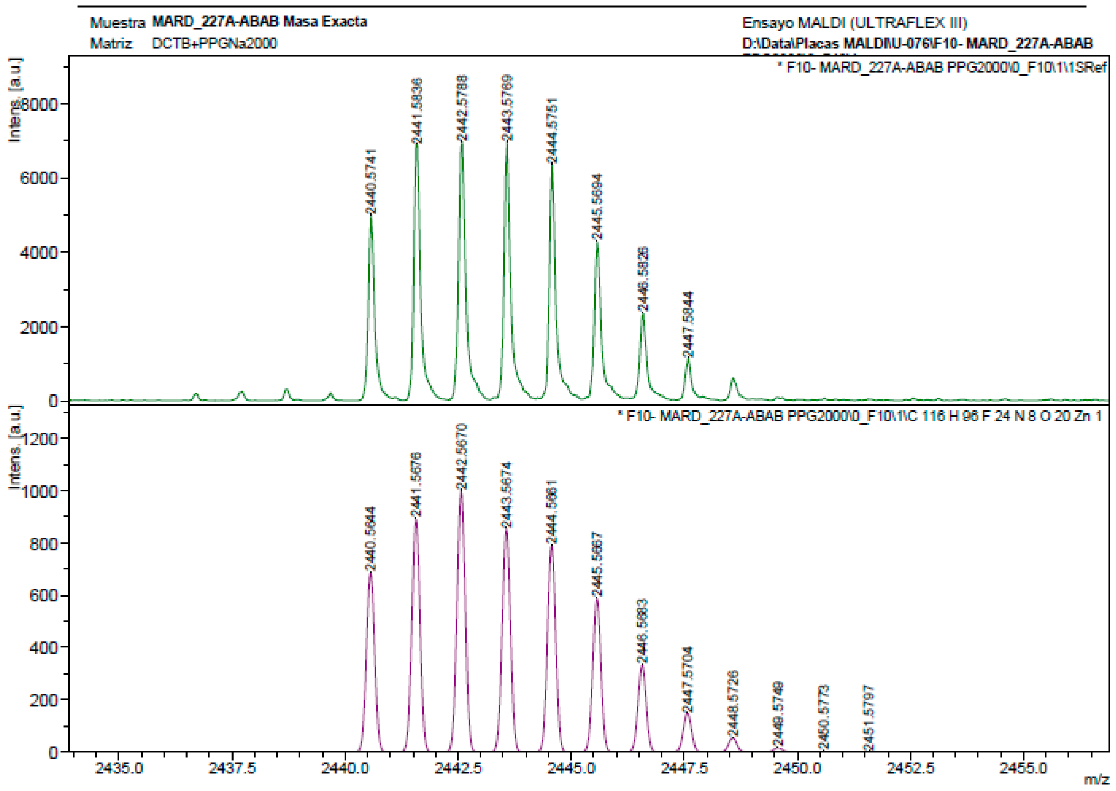

^{13}C NMR in $CDCl_3$:

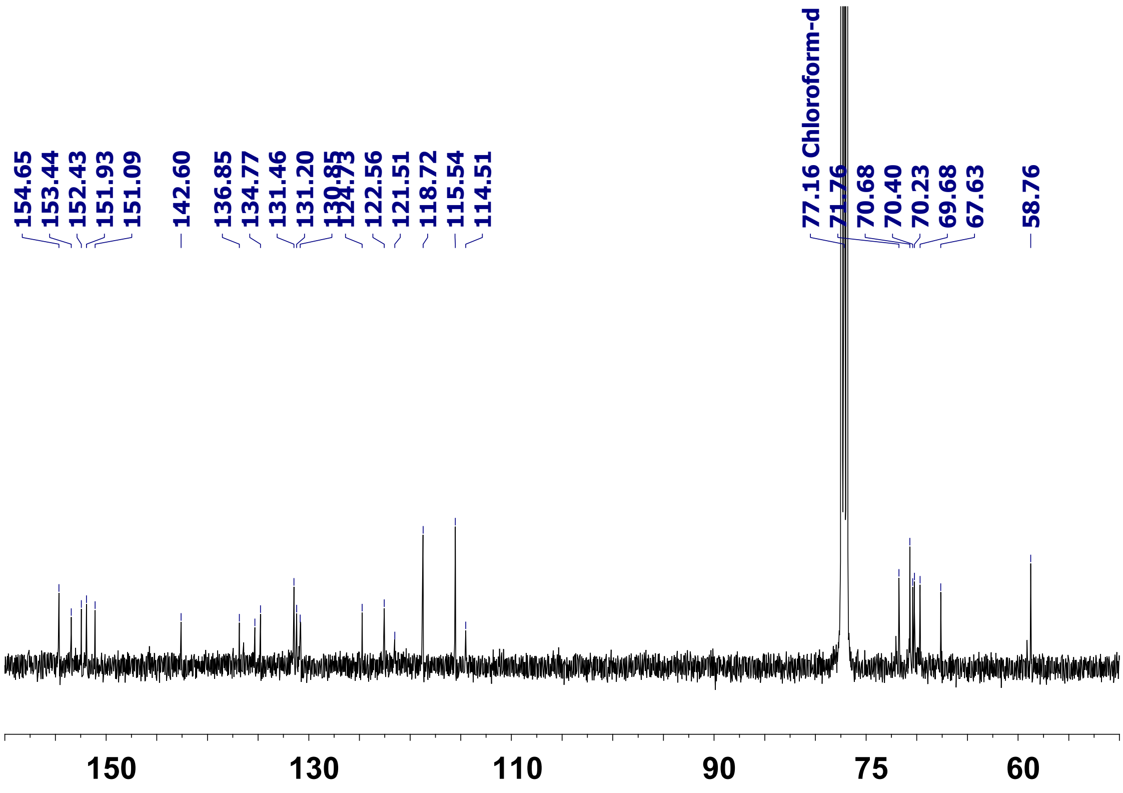

A3B Zn(II)Pc 1:

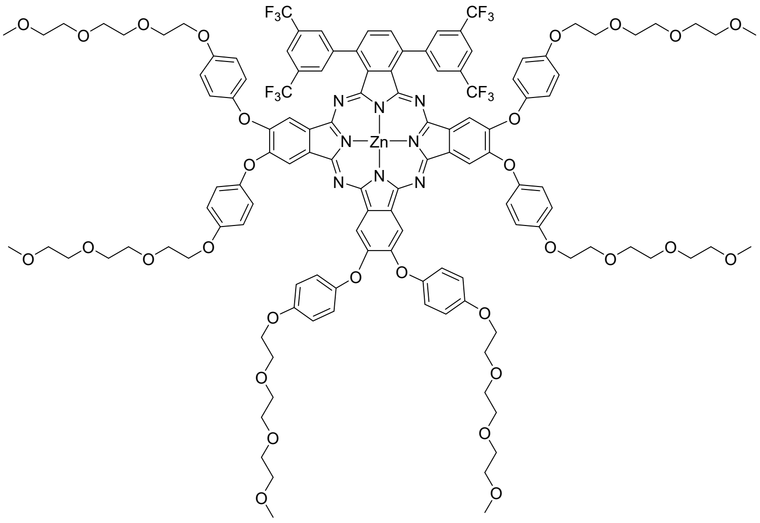

MS and HR-MS:

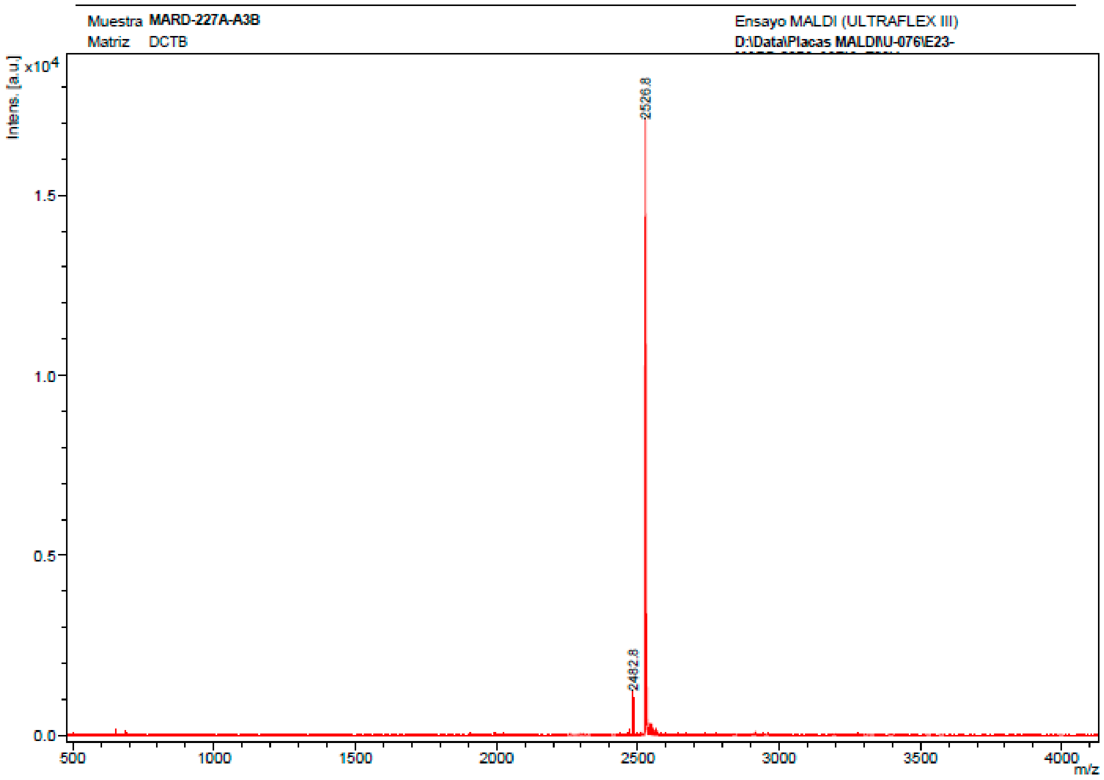

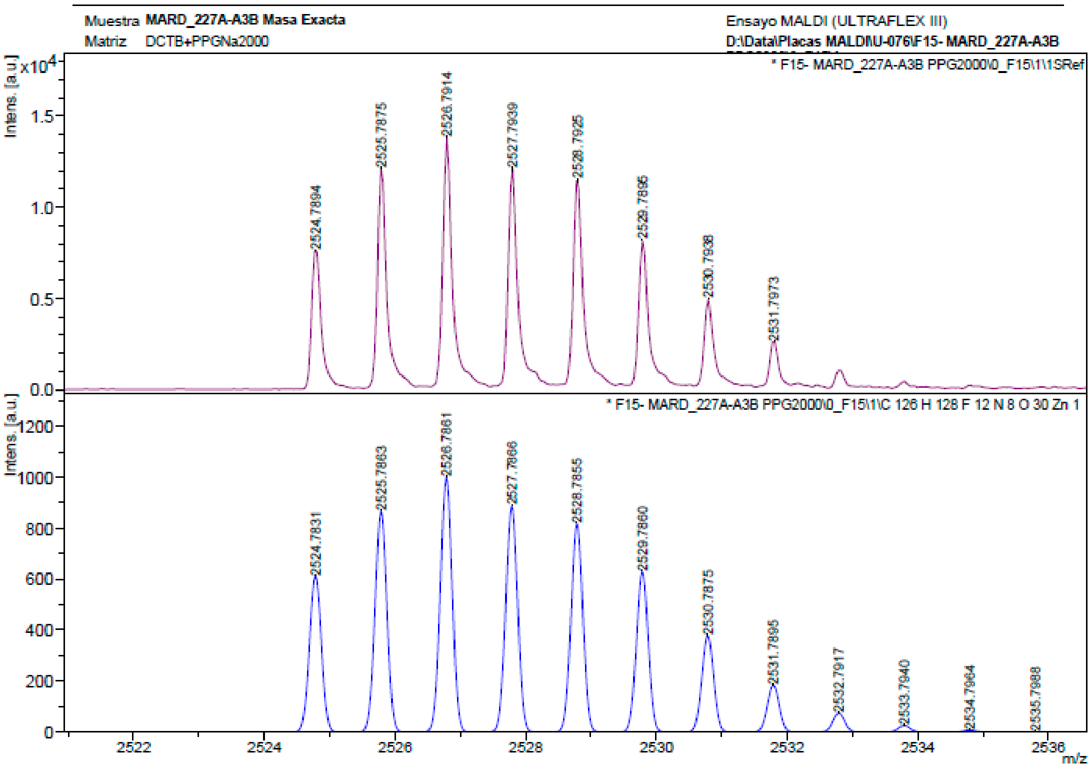

^{13}C NMR in THF-d$_8$:

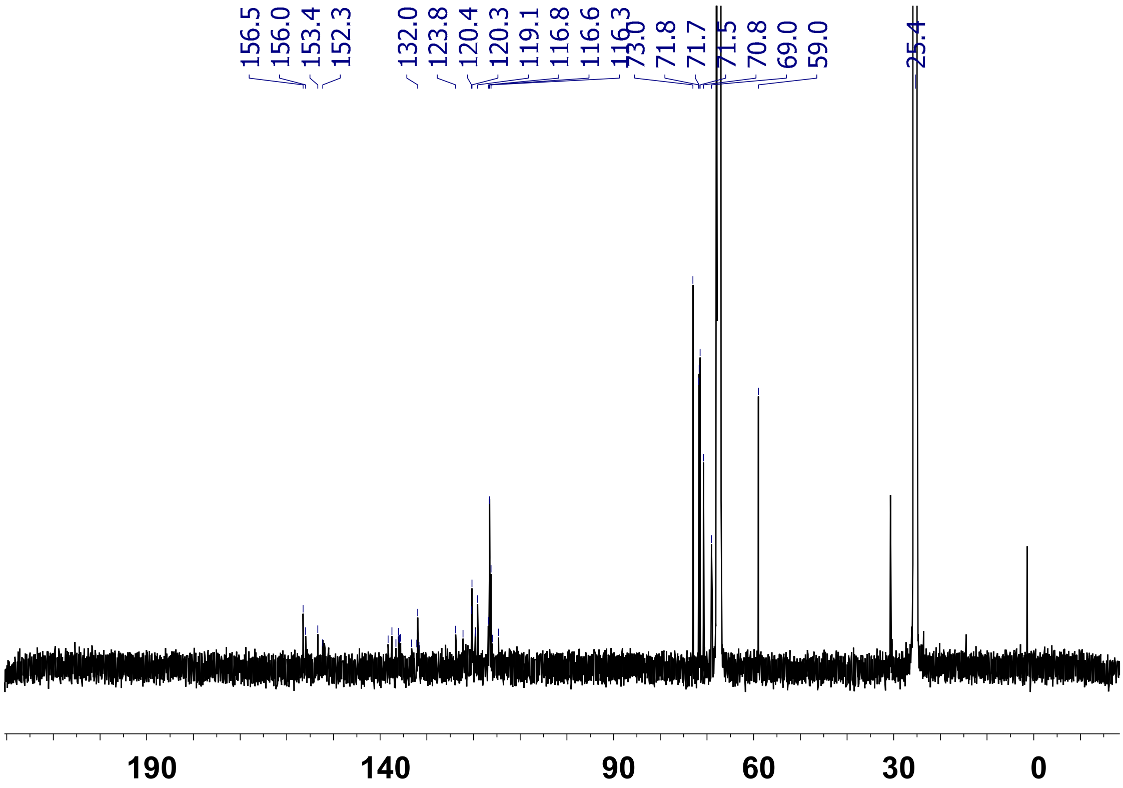

A$_4$ Zn(II)Pc 1:

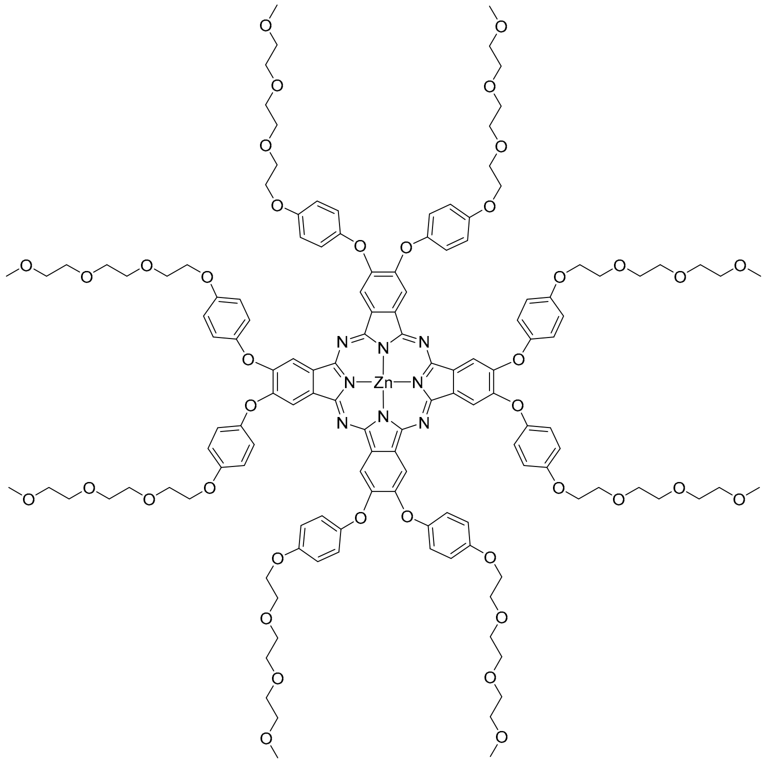

MS and HR-MS:

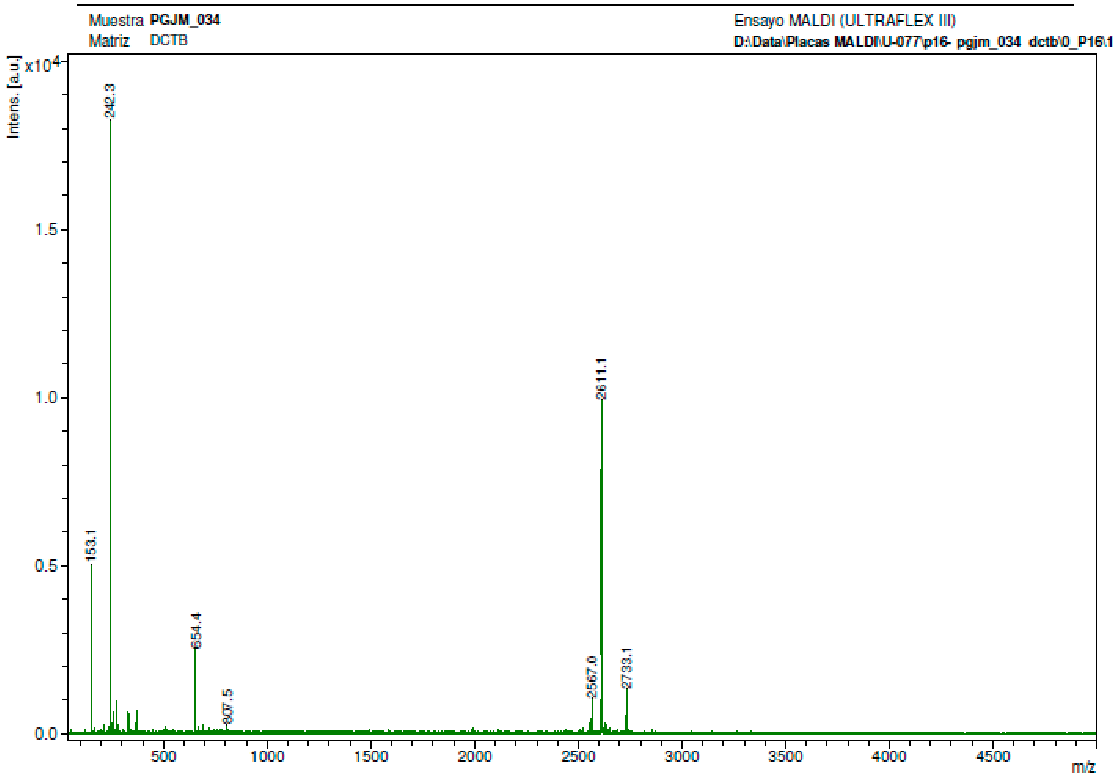

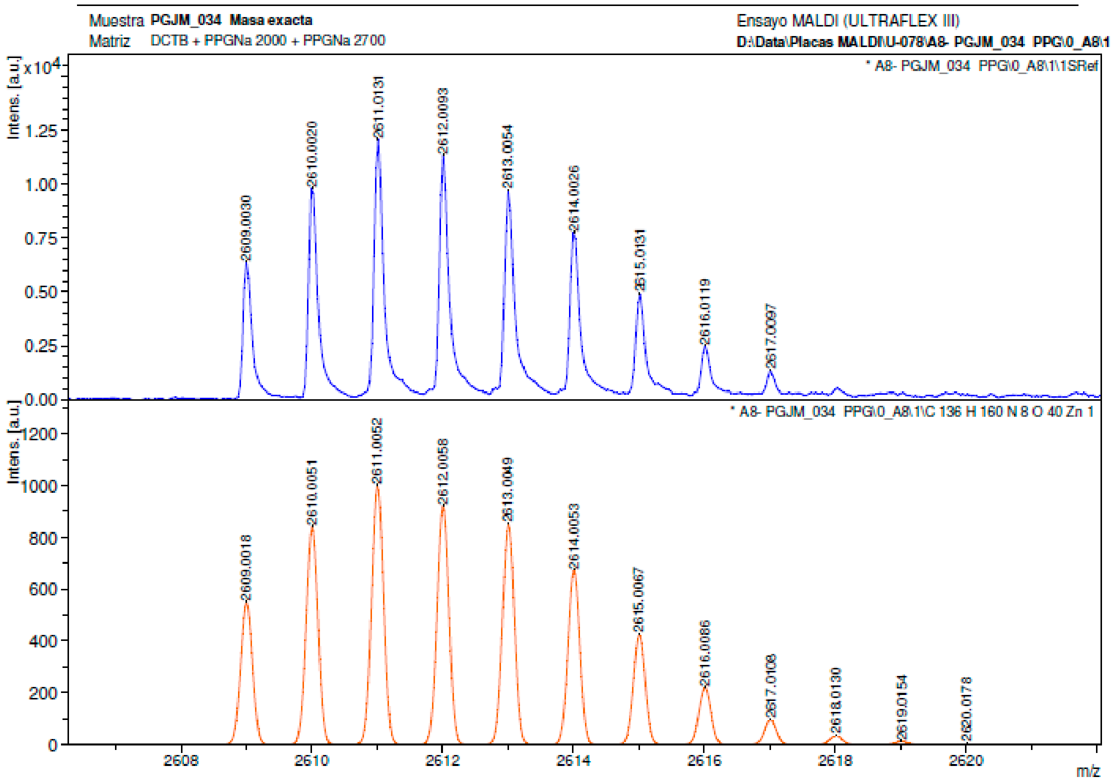

^{13}C NMR in DMSO-d$_6$:

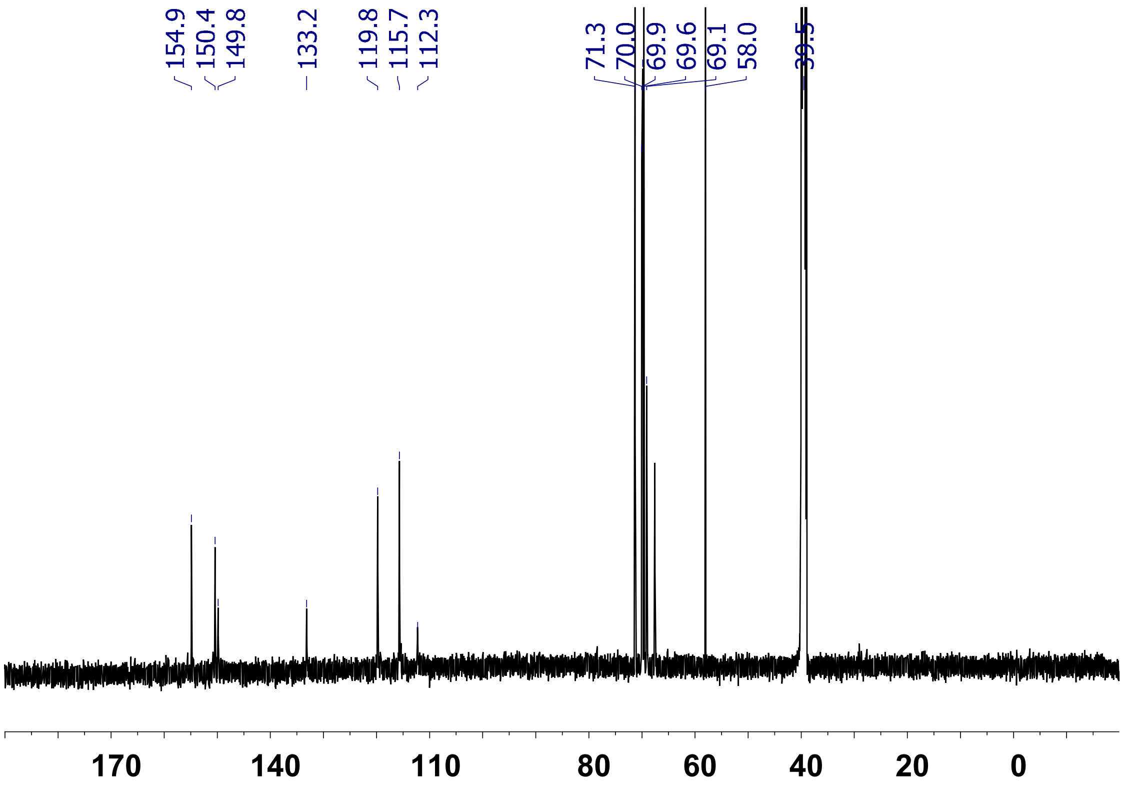

Supplement: Supplementary file 1 [file molecules-25-00213-s001.pdf]
